# MXene-integrated quantum dots: an innovative tool for next-generation healthcare applications

**DOI:** 10.1039/d6na00252h

**Published:** 2026-09-04

**Authors:** Subhendu Chakroborty, Nibedita Nath, Dinesh Kumar Pati, Sunita Singh, Ranjita Priyadarshini Biswal, Swarna P. Mantry, Manish Kumar, Rajib Padhee, Karunesh Tiwari, Chandra Chakraborty

**Affiliations:** a Environmental Science Laboratory, School of Applied Sciences, Kalinga Institute of Industrial Technology (Deemed to be University) Bhubaneswar-751024 India subhendu.cy@gmail.com; b Department of Chemistry, DS Degree College Laida Sambalpur Odisha India nibeditanath.mami@gmail.com; c Newton Higher Secondary School of Science Debaipali Sambalpur Odisha 768200 India; d IES College of Education, IES University Bhopal Madhya Pradesh 462044 India; e Department of Basic Science, Department of Chemistry, KIIT Polytechnic Bhubaneswar India; f Department of Chemistry, Sir M. Visvesvaraya Institute of Technology Bengaluru Karnataka 562157 India; g KIET School of Pharmacy, Krishna Institute of Engineering & Technology (KIET) Ghaziabad Delhi-NCR Uttar Pradesh India; h School of Physics, Sambalpur University Jyoti Vihar, Burla Odisha 768019 India; i Department of Physics, Mai Nefhi College of Science, Eritrean Institute of Technology Mai Nefhi Eritrea tdrkarunesh@gmail.com; j Department of Allied Sciences, Graphic Era (Deemed to be University) Clement Town Dehradun 248002 India

## Abstract

Due to their superior physical and chemical characteristics, MXene quantum dots (MXQDs) are regarded as outstanding zero-dimensional nanomaterials. The excitation-dependent emission properties, photostability, biocompatibility and dispersion stability of these QDs make them potential candidates for solving biomedical and environmental problems. Owing to their remarkable properties and biocompatibility, MXQDs represent a new generation of smart nanosystems used for cancer diagnostics and treatment, imaging, sensing, tissue engineering, and drug delivery. However, there are many problems related to their biodegradability, photoluminescence, long-term toxicity, and biodistribution properties, which need to be carefully considered. This review focuses on the current developments of MXQDs in healthcare applications and also on the current trends and future perspectives.

## Abstract 2

MXene-integrated quantum dots combine tunable photoluminescence, rich surface chemistry, and biocompatibility for advanced bioimaging, biosensing, drug delivery, cancer therapy, and tissue engineering applications.

## Graphical abstract

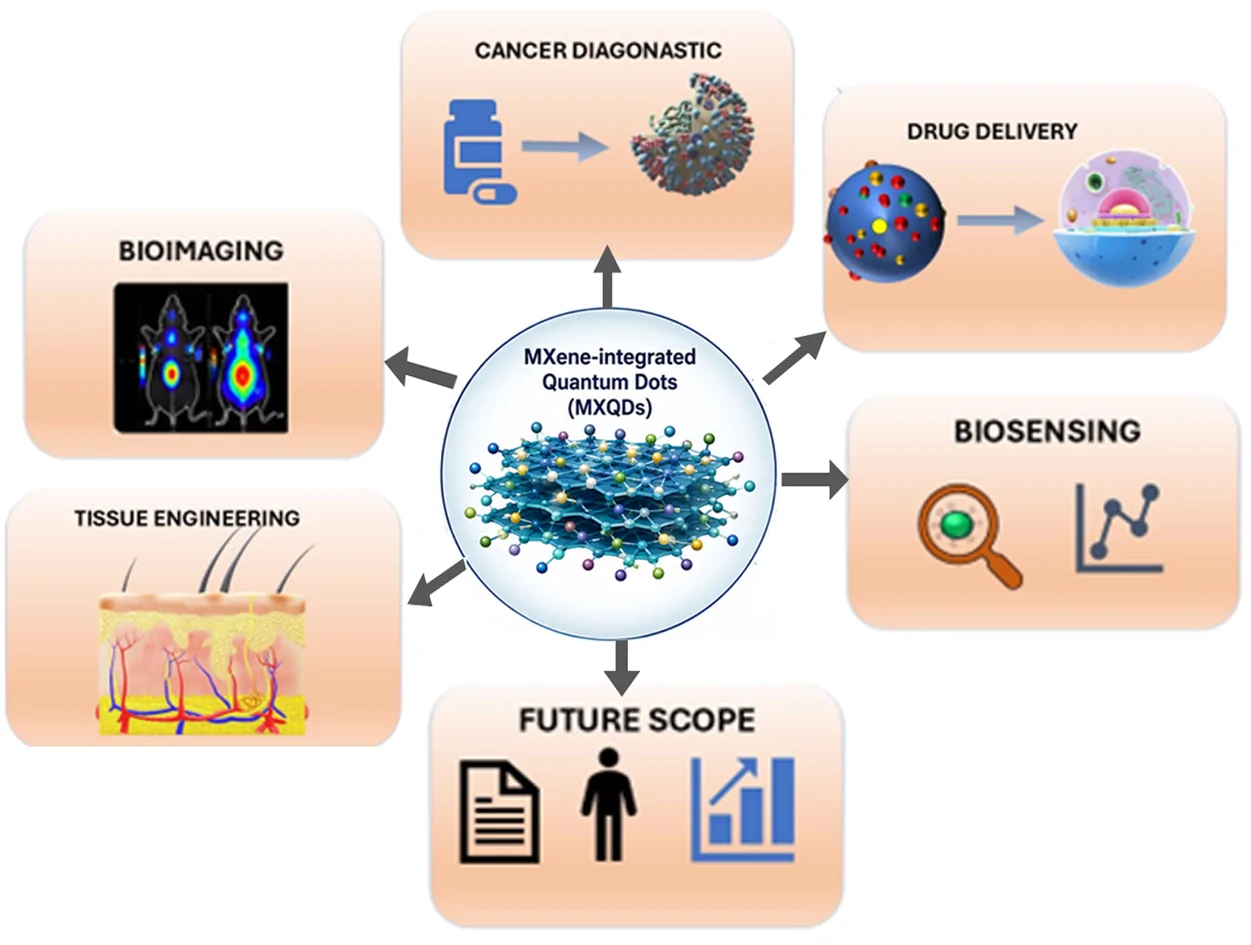

## 1. Introduction

Innovation in healthcare development started with the incorporation of two-dimensional (2D) nanomaterials (NMs) in biomedical applications, which further enhanced the technology, enabling the development of smart and portable devices for personal health monitoring. These materials are distinguished by their flexibility, functionalized surfaces and high surface-to-volume ratio. From initial discovery to targeted developments, the evolution of these materials in healthcare follows a path that directly affects their viability and efficacy in healthcare applications to address specific restrictions or obstacles.^1,2^

2D NMs such as graphenes are essential in biosensing and biomedical research because of their remarkable mechanical strength, high electrical conductivity, and vast surface area.^3,4^ MXenes (M_*n*+1_X_*n*_T_*x*_), also known as 2D transition metal carbides and nitrides, exhibit remarkable qualities for medical research and applications. For example, their hydrophilic surface provides strong colloidal stability and antibiofouling qualities.^5,6^ The metal-like conductivity of MXenes makes them valuable materials for a variety of applications, including wireless antennas, electrochemical sensors, and actuators for soft robotics.^7^ In biomedical applications, their chemically active surface subsequently offers control over a number of crucial elements, including drug administration, controlled release, external functionalization for biosensing, theranostics, and cell–material interactions.^8^

The Nobel Prize in Chemistry was given to quantum dot (QD) technology in 2023 for its exceptional contributions to materials science and biomedicine. Elements from groups II–VI or III–V in the periodic table make up QDs, which are nanoscale particles with a typical particle size of 1–10 nm.^9,10^ The quantum confinement effect and small size of QDs^11^ endow them with special fluorescence characteristics that make them resistant to photobleaching. They have a lot of potential for use as fluorescent probes in biomedical applications, as their size can be tuned to achieve the desired fluorescence hue. Known for their structural integrity, QDs have a large specific surface area^12^ and the inherent ability to pass through cell membranes with ease.^13^ QDs offer hope for advanced nanotechnology. QDs have been used more and more in the fields of pharmacy and medicine in recent years.^14^

In 1981, Ekimov and Onushenko introduced quantum dots (QDs), commonly referred to as nanoscale semiconductor crystals, in a glass matrix.^15,16^ The first biological imaging application of QDs has been reported in 1998.^17^ Since then, the field of quantum dots (QDs) has expanded significantly, and currently, it has applications in solar cells, photovoltaic devices, computation systems, biomedical imaging devices, light-emitting diodes (LEDs), and photodetectors, among other applications. Since Alexei Ekimov's accidental discovery of quantum dots (QDs) in the 1980s, the field of nanotechnology and nanoscience has paid close attention to these particles, which typically have sizes between 2 and 10 nm. With remarkable qualities like fluorescence, visible light absorption, quantum confinement effects, abundant surface-active sites, and the multiexciton phenomena,^18–20^ QDs show great promise as materials for various applications.^21–23^ Biosensing, optoelectronics, energy storage, catalysis and medical applications are among the targeted sectors where MXQD publications have grown significantly to date. Rather than covering every aspect of other intriguing uses, we focus on the current research state of MXQDs in healthcare applications in this review. Additionally, an overview of MXQD research developments from synthesis and features to limitations and challenges is provided. Lastly, a brief discussion of their limitations, challenge and perspective on the biomedical sector is provided. We believe that this analysis will help to direct the rational development of high-performance MXQD materials for next-generation healthcare applications. To this day, interest in MXQDs research continues to grow, and its application in the biomedical industry is gradually gaining traction.

Therefore, we concentrate on the latest developments in MXQDs in this review, giving an organized review of their synthesis, unique features, surface engineering and biological significance. We examine current developments in the application of MXenes to boost the functionality of MXQD-based devices in the direction of more useful strategies for the upcoming generation of medical devices. Lastly, we present a critical review of the present issues, constraints, and opportunities for the future development of MXQDs for wearable POC applications, focusing on their vast sensing potential that has not yet been explored. Our analysis highlights the recent developments in healthcare and offers new perspectives on the identification of MXQDs. Therefore, it will offer a direction and a point of reference for the synthesis of new MXQDs and the development of high-performance materials based on MXQDs.

Previous reviews on MXQDs suffer from major literature gaps and limitations regarding their evaluation of synthesis, properties, and limited biomedical uses. This review provides guidance for researchers to close the gaps between conventional medical methods and the versatile biomedical platform required for applications in the next generation of healthcare.

## 2. MXene-derived quantum dots

Researchers around the world have been impressed by the emergence of MXenes and their nanostructures with intriguing physical and chemical properties such as hydrophilicity, multimodal sensing/imaging capabilities, photo-thermal stability, large surface area, and thermal/electrical conductivity.^24–26^

Gogotsi's group first developed MXenes, 2D metal carbides/nitrides, in 2011 by selectively etching the “A” layers from MAX catalysts.^27,28^ Because of their desirable physical and chemical characteristics, such as photothermal stability, conductivity, hydrophilicity, and ease of surface modification, these innovative 2D nanomaterials have found extensive applications in various fields, including biomedicine, sensing, supercapacitance, and catalysis.^29–33^ MXene blocks become MXQDs when they are reduced in size to 10 nm, maintaining some of the benefits of their two-dimensional parent structure but exhibiting a number of distinct physical and chemical characteristics.^34,35^

MXQDs have a large specific surface area than that of MXene blocks, better biocompatibility, more tunable characteristics, and ability to hybridize with other nanomaterials more easily, and they are simpler to dope or functionalize.^36–38^ For example, MXQDs exhibit better electrical and optical characteristics than MXene nanosheets due to their quantum confinement and edge effects. Their high attenuation coefficient and dielectric constant allow them to readily split into electrons and holes. Furthermore, MXQDs are better choice for the detection of toxic chemicals in organisms since they have far lower cytotoxicity than MXenes. MXQDs also have long-term stability, size dependency, and tunable photoluminescence (PL). Compared to MXenes, these benefits give MXQDs more opportunities in the sensing domain.^36,39,40^ Additionally, MXQDs have outstanding stability, superb photoluminescence, and great biocompatibility. MXQDs are incredibly useful instruments for controlling immune function in the field of immunomodulation because they provide precise control over immune responses. In order to help treat autoimmune illnesses, allergies, and inflammatory disorders, they can be designed to either activate or decrease immune responses.^36,41^ Furthermore, MXQDs have demonstrated promise in cancer treatment as effective drug delivery systems. Therapeutic medicines like immune checkpoint inhibitors or chemotherapeutic medications can be loaded onto their large surface area and then precisely targeted to tumor locations.^41,42^ The examples of the biomedical uses of MXQDs are included in Table 1.

**Table 1:** Some notable biomedical applications of MXQDs^a^

MXQDs	Applications	References
Ti_3_C_2_	PTT	42
Ti_2_N	PA + PTT	43
Ti_3_C_2_	PL imaging	44
Mo_2_C	Bioimaging and PTT	45
V_2_C	Tumor destruction	46
Ti_3_C_2_	Cellular imaging probe	47
Ta_4_C_3_T_*x*_	Transplant vasculopathy	48
Ti_3_C_2_T_*x*_	Nanomedicine	49

a PTT: photothermal therapy; PA: photoacoustic imaging; and PL: photoluminescence.

M_*n*+1_X_*n*_T_*x*_ is the formula for MXenes, a broad class of 2D materials that includes transition metal carbides, carbonitrides and nitrides. Here, M stands for transition metals like Ti, V, Mo, and Nb, X is carbon/nitrogen, *n* can be 1–3, and T_*x*_ represents surface terminations such as fluorine, oxygen, and hydroxyl.^49,50^ As a result, MXenes are varied, have rich chemical compositions, and are constantly growing.^51^ –O, –F, and –OH terminations shift energy gaps and absorption in TiCT MXQDs; O provides the biggest gap, whilst OH/F shifts absorption toward visible/NIR and significantly alters exciton binding up to 75% of the gap.^52^ Size reduction typically enhances bandgap (blue shift) by quantum confinement.^53^ Rich valence states, an adjustable bandgap, and outstanding photothermal properties are just a few of the chemical and physical characteristics that might result from the varied elemental compositions and are important for tumor therapy.^54,55^ Additionally, MXenes are predicted to substitute noble metals in a variety of applications due to their similar catalytic properties.^56–58^

### 2.1. Synthesis of MXQDs

Since MXQDs have developed so quickly, numerous synthesis techniques have been developed to satisfy the demands of high yield, high rate, and simple workup process. The majority of 2D-MXQDs can be prepared from their native layered MXenes and synthesized directly by reactions. Generally speaking, there are two primary categories of synthesis methods of MXQDs: top-down and bottom-up approaches.^59^ The chosen synthetic techniques may influence the size, chemical groups, and structures of MXQDs. Table 2 presents the pros and cons of various methods of synthesis of MXQDs.

**Table 2:** Pros and cons of various methods of synthesis of MXQDs

Synthesis method	Pros	Cons
Hydrothermal	Facile; high yield; tunable size/shape *via* temperature	Requires high-pressure vessel; potential for oxidation
Solvothermal	Similar to hydrothermal; uses organic solvents for surface modification	Involves complex solvent handling
Ultrasonication	Green; eco-friendly; rapid fragmentation	Lower yield compared to chemical methods; lower crystallinity
Ball-milling	Simple; suitable for scaling up, dry processing possible	Potential for edge impurities; wide size distribution
Electrochemical	High purity, excellent size control, easy to operate	Less conventional; harder to optimize
Pyrolysis	No fluorine content, less time needed	High temperature needed, composite product is formed
Molten salt	No fluorine content, control morphology	High temperature needed, composite product is formed

These techniques are highly valued for preserving the original materials' structural characteristics, such as crystalline domains and particular edge configurations, but they frequently call for severe reaction conditions, specialized tools, and a large energy input. Additionally, they frequently generate QDs with wide size distributions and lower quantum yields, which can cause challenges with scalability, regulatory approval, and batch-to-batch reproducibility, especially under conditions in which strict control over surface chemistry and biocompatibility is important for biomedical applications.^60^ QDs synthesized using bottom synthesis techniques such as hydrothermal processing, microwave-assisted pyrolysis, thermal decomposition, and template-directed assembly from molecular precursors provide flexibility to adjust the particle size, surface functional groups, and optical features. More economical and sustainable manufacturing methods are made possible by the flexibility in precursor selection, which ranges from simple organics to biomass. However, these methods frequently result in heterogeneous populations that require thorough purification to eliminate the remaining byproducts, which may hinder reproducibility and raise questions about clinical scalability.^61^ Productivity, homogeneity, and scalability issues are starting to be addressed by emerging technologies such as continuous flow reactors, automation, hybrid synthesis methods, and AI-driven process optimization.^62^

The main problem in the fabrication of MXQDs is the dimensional reduction of transition metal carbide/nitrides into stable MXQDs. This includes changes in the chemical composition as well as photophysical characteristics including edge effects, quantum confinement, and increased surface area.^63^ Thus, it is important to understand the various synthesis methods and their requirements in order to benefit from the significant impact that the synthetic parameters have on determining the characteristics of the final product.^38^

There are two types of synthesis techniques: top-down and bottom-up. To create oxygen-containing MXene nanosheets, the bulk precursor molecules are broken down into 2D nanosheets in the top-down approach, which is mostly chosen. These subsequently operate as locations for the development of flaws and chemically reactive sites.^38^ Furthermore, various cutting procedures can readily break down the multilayer structure into smaller equivalents, which ultimately results in the development of a quantum dot.^64^ According to the findings, the characteristics of the quantum dots can also be significantly impacted by the various methods used to break down the MXene sheets. Hydrothermal/solvothermal, electrochemical cutting and ultrasonication are examples of such methods.^45,65,66^ The characteristics of the generated MXQDs are determined by a number of factors including temperature, concentration, etching time, and etchant type.^65,66^

Ultrasonication is a safe, environmentally friendly technique. A multilayer material is altered by cavitation, reverberation, and solvent acoustics. The size, shape, surface chemistry and general condition of the resultant MXQDs are greatly impacted by the power, ultrasonication time, intensity, and the type of solvent. When compared to other high-energy approaches, this approach uses less energy.^67^ Ultrasound-assisted synthesis uses organic solvents such as dimethyl sulfoxide (DMSO), dimethyl formamide (DMF), *N*-methyl-2-pyrrolidone (NMP), and tetrabutylammonium hydroxide (TBAOH).^68^ MXenes are generated by fluoride etching, and then, the mixture of MXenes and ammonia was ultrasonicated to create nitrogen-doped MXenes.^69^

One-step ultrasonic exfoliation method was used by Zhang *et al.* to produce fluorescent MXQDs. These QDs show an outstanding fluorescence quantum yield of 7.7% and also exhibit high selectivity and sensitivity for the detection of Fe^3+^ in blood and saltwater. The QDs have significant benefits due to their excitation-dependent and pH-independent emission properties.^70^ Currently, MXQDs having a moderate QY of ≈10–28% are biocompatible and photostable, and are useful for multicolored cellular imaging. However, MXQDs are still primarily visual emitters and in their early stages when compared to mature NIR-emitting semiconductor QDs and carbon dots that had already offered deep tissue and entire body imaging.^71^Table 3 shows the comparison of the QY of various QDs with that of MXQDs.

**Table 3:** Comparison of the QY of various QDs

QDs	QY	Emission regime	Toxicity	Photostability	Biodegradability	Targeting/translation	Application	Reference
Semiconductor	High	Visible-NIR-II	Highly toxic	Excellent	Non-biodegradable	Clinical use inhibited	Deep imaging	72
Carbon	Usually high	Visible-NIR	Less toxic	Excellent	Biodegradable	Translation is at an advanced stage	*In vivo* imaging	73
MXene	∼10–28%	Visible	Low toxicity	Excellent	Biodegradable	Preclinical stage	Cell imaging	71

Cao *et al.* used a hydrothermal process with a V_2_C nanosheet precursor to create V_2_C QDs. V_2_C QDs were single-layer atomic crystals, as evidenced by their average thickness of 0.8 nm.^74^ Ai *et al.* successfully produced MXQDs *via* a one-step hydrothermal process having amino functional groups. MXQDs have exceptional photostability, vivid blue fluorescence (FL), high quantum yield and a restricted lateral size distribution.^75^

MXQDs doped with nitrogen and chlorine have been developed using the electrochemical etching method. Here, the electrolyte environment was generated using tetramethylammonium (TMA) hydroxide and NH_4_Cl, which then interacted with Ti and C layers. The applied electric potential allowed the C layers at the defect boundaries to break apart by interacting with electrolyte ions, while the Cl and Ti ions form Ti–Cl termination. This favored the formation of N–Ti_3_AlC_2_ QDs from the bulk Ti_3_AlC_2_ MAX-phase working electrodes.^76^

Numerous advantages including easy handling, abundant raw material supply, less equipment requirements, large-scale production, improved control of morphology, and less duration are provided by MXQDs produced by a top-down synthesis method. Compared with the bottom-up synthesis method, the MXQD products tend to be more pure, reliable, and efficient.^77^ The disadvantages of top-down procedures include the use of corrosive acids, like HCl, HNO_3_ and HF_3_, quick oxidation, complex post-treatments, and harsh conditions.^78^ Alternative fluoride-free methods were developed to address the risks related to HF. For instance, the synthesis of fluorine-free QDs from MAX precursors was made possible using a technique that combined ultrasonication with tetrabutylammonium hydroxide (TBAOH) for the selective removal of Al layers.^79–82^ However, structural flaws, dangling bonds, and leftover fluoride functional groups are frequently introduced during HF etching, which deteriorates the photoluminescence characteristics and reduces the suitability of QDs for optoelectronic applications.^83^ Additionally, HF poses a risk to lab scientists and industrial researchers due to its extreme toxicity, corrosiveness, and difficulty in safe handling.^84^ Due to these constraints, scientists are investigating safer, quicker, and more ecologically friendly HF-free methods.

Pulsed laser irradiation in liquids (PLIL) is one such promising substitute. The main advantages of PLIL include its capacity to generate high-purity, ligand-free QDs without the use of hazardous chemical precursors, which makes it especially appropriate for applications in optoelectronics and bioimaging.^85,86^ Green, HF-free laser ablation in water for the synthesis of QDs and their uses in photocatalysis^87,88^ and lasing^89^ have been reported in a recent work. Shivappanayaka's research group synthesized extremely fluorescent V_2_C QDs from the MAX phase directly using pulsed laser ablation in a binary solvent environment without using HF by a green method.^90^

When compared to top-down methods, the bottom-up strategy is more effective, less expensive, and less time-consuming. Among the techniques falling under this category are pyrolysis, chemical vapor deposition, and electrodeposition. Because it begins at the molecular level and makes its way up, the synthetic procedure under discussion guarantees a more uniform composition and fewer defects in the finished product. Although it is a very costly, energy-intensive, and advanced technology, it allows for better control and enhanced structure. Research on bottom-up strategies has been constrained by these issues, which inspires researchers to investigate the methods.^91–93^

Wang *et al.* used pyrolysis to create a Mo_2_C QDs/carbon polyhedron composite. Molybdic acid, 2-methylimidazole and zinc acetate were used as precursors in the pyrolysis of Mo/ZIF-8, which was carried out for two hours at 700 °C in an air atmosphere. The zinc species was ultimately eliminated with the use of acidic etching. The average diameter of the generated Mo_2_C QDs was determined to be 4.5 nm. The lattice spacing of QDs was found to be 0.24 nm, which is in good agreement with the cubic-Mo_2_C (111) plane.^94^ It makes sense to construct MXQDs using bottom-up approaches, but mono-dispersed species, moderate conditions, good crystallinity, and low-toxicity precursors have to be taken into account. Because of its comparatively straightforward operation, the MXQDs may be prepared by a one-pot top-down synthesis method and used in the future. Although there has not been much study on the bottom-up synthesis of MXQDs, this study is encouraging and merits further consideration.^38^

Ti_3_C_2_ MXQDs were first produced by etching the MAX phases and then exposed to microwave-assisted irradiation for five minutes. In contrast to alternative bottom-up methods, our microwave-assisted synthesis considerably shortened the reaction time for the production of Ti_3_C_2_ MXQDs.^95^

### 2.2. Properties of MXQDs

The energy levels and electronic transitions of MXQDs are determined by their electronic band structure, which can affect their electrical and optical characteristics. MXQDs feature intriguing electrical properties and distinctive band topologies. These QDs typically have one or a small number of layers and are smaller than 10 nm. Effective light absorption in the visible, near-infrared, and ultraviolet spectra is made possible by the ability to alter the electronic band size of MXQDs.^38^

The electrical characteristics of MXenes are crucial. Regarding the MXQDs, their small-size effect should make their electronic qualities more appealing. In theory, the d-electrons from the ‘M’ atoms give the MXQDs their high electron density and metallic conductivity at the Fermi level (EF).^96^ According to experiments, MXene sheets exhibit better electric conductivities than carbon nanotubes and reduced graphene oxide materials, and they are comparable to multilayered graphene resistance in the range of 22–339 U.^97,98^ Nanosized MXQDs are expected to be better for electron transfer than their stacked counterparts because the electronic conductivity can be adjusted based on the composition and terminated groups of MXQDs. It has also been found that the number of layers and the presence of surface groups increase resistance.^98,99^

Among the advantages that MXenes offer over traditional materials are exceptional conductivity, tunable surface terminations, good carrier mobility and a flexible work function. Because of this properties, MXQDs are used in various applications, including catalysis, photocatalysis, and perovskite solar cells.^100,101^ High conductivity and carrier mobility of MXQDs improve charge transfer in perovskite solar cells.^101^ MXQDs have superior catalytic properties due to active edge sites and excellent charge transport.^100^ Furthermore, the excellent charge separation and carrier mobility of MXQDs enable the sensitive detection of glutathione.^102^

MXQDs have demonstrated outstanding photothermal characteristics, particularly titanium carbide (Ti_3_C_2_) MXQDs. Excellent near-infrared photothermal characteristics for the treatment of cancer were demonstrated by the fluorine-free production of Ti_3_C_2_ MXQDs, and 52.2% photothermal conversion efficiency was demonstrated by the produced Ti_3_C_2_ quantum dots.^43^ The photothermal characteristic of Ti_3_C_2_ MXQDs makes them appropriate for a number of applications, such as phototherapy and biosensing procedures. Because of their exceptional photothermal efficiency, hydrophilia, and thermal stability, they have been employed as phototherapy reagents. Furthermore, Ti_3_C_2_ MXQDs have demonstrated tremendous promise in the bioanalytics sector.^103^

Because of their distinct composition and structure, MXQDs have interesting fluorescence features. One important characteristic of MXQDs for biosensors and diagnostics is their fluorescence property. Several tuning techniques are used to further improve the fluorescence property of MXQDs. The tuning of fluorescence in MXQDs can be achieved through strain engineering, doping and external conditions.^104^ These methods control the fluorescence emission from the MXQDs in terms of wavelength, intensity, and stability. Excellent fluorescence abilities have been demonstrated by MXQDs, making them suitable for use in optoelectronic devices, imaging, and sensing.^105^Table 4 shows the various properties of MXenes compared to the other 2D materials. These unique properties make MXQDs special compared to the other 2D materials.

**Table 4:** Various properties of MXenes

Properties	MXenes	Other 2D materials	Ref
Synthesis	Highly scalable	Less scalable	106
Stability	Highly stable	Less stable	107
Mechanical strength	High elastic constant	Low elastic constant	108
Hydrophilicity	Highly hydrophilic	Mostly hydrophobic	109
Conductivity	High	Semi-conductive/h-BN is insulator	110
Electronic	Tunable	Less tunable	111
Surface modification	Tunable	Limited	112

The lifespan of QDs is greatly influenced by their surface chemistry, which includes the type and stability of their surface ligands. Although ligands shield QDs from oxidation and aggregation, they can deteriorate over time, reducing its efficiency and fluorescence intensity. In order to stabilize quantum dots, surface ligand engineering and selection are essential. The solubility of QDs can be improved, and aggregation can be avoided *via* ligand exchange with strong molecules like thiols, silanes, or zwitterionic chemicals. Furthermore, an additional layer of defense against oxidation and photobleaching is offered by core–shell architectures like CdSe/ZnS.^113^

## 3. MXQDs for healthcare applications

QDs have emerged as highly promising materials for biomedical applications because of their many advantageous characteristics, including biocompatibility, fluorescence, solubility in water, and low toxicity. They are now a well-liked choice for a range of biomedical uses, such as antibacterial particles, therapeutic genes, drug nanocarriers, and photosensitizers. Their capacities must be demonstrated in the development of theragnostic nanomedicine, cellular and bacterial bioimaging, and multifunctional diagnostic levels. They will soon be applied in intracellular and extracellular research to identify different types of cancer cells, therapeutic processes and atomic components of diseases.^114^ This review highlights the recent biomedical applications of MXQDs.

### 3.1. MXQDs for biosensing

Because of its great sensitivity, numerous applications, inexpensiveness, and low dosage, biomedical sensing technology quickly supplanted the traditional method that involved only one type of analysis for detection. Technology advancements have made it possible for sensing detectors to track biological cells *in vivo*, which would significantly improve our study techniques for a number of alterations in cell growth, metabolism, and cancer. In recent years, there has been extensive research on tumor detection, and it has been discovered that using modified nitrogen-doped MXQDs may help to trace and identify tumor cells.^44^

Researchers are interested in exploring the use of MXQDs in future-generation diagnosis due to their superior physical, chemical, and optoelectronic characteristics as well as their extensive functionalization and tunability windows.^115,116^ They can be used as optoelectronic probes for sensor development, protecting human health and enabling major advancements in the healthcare industry through early disease diagnosis.^115^

**Fig. 1:**
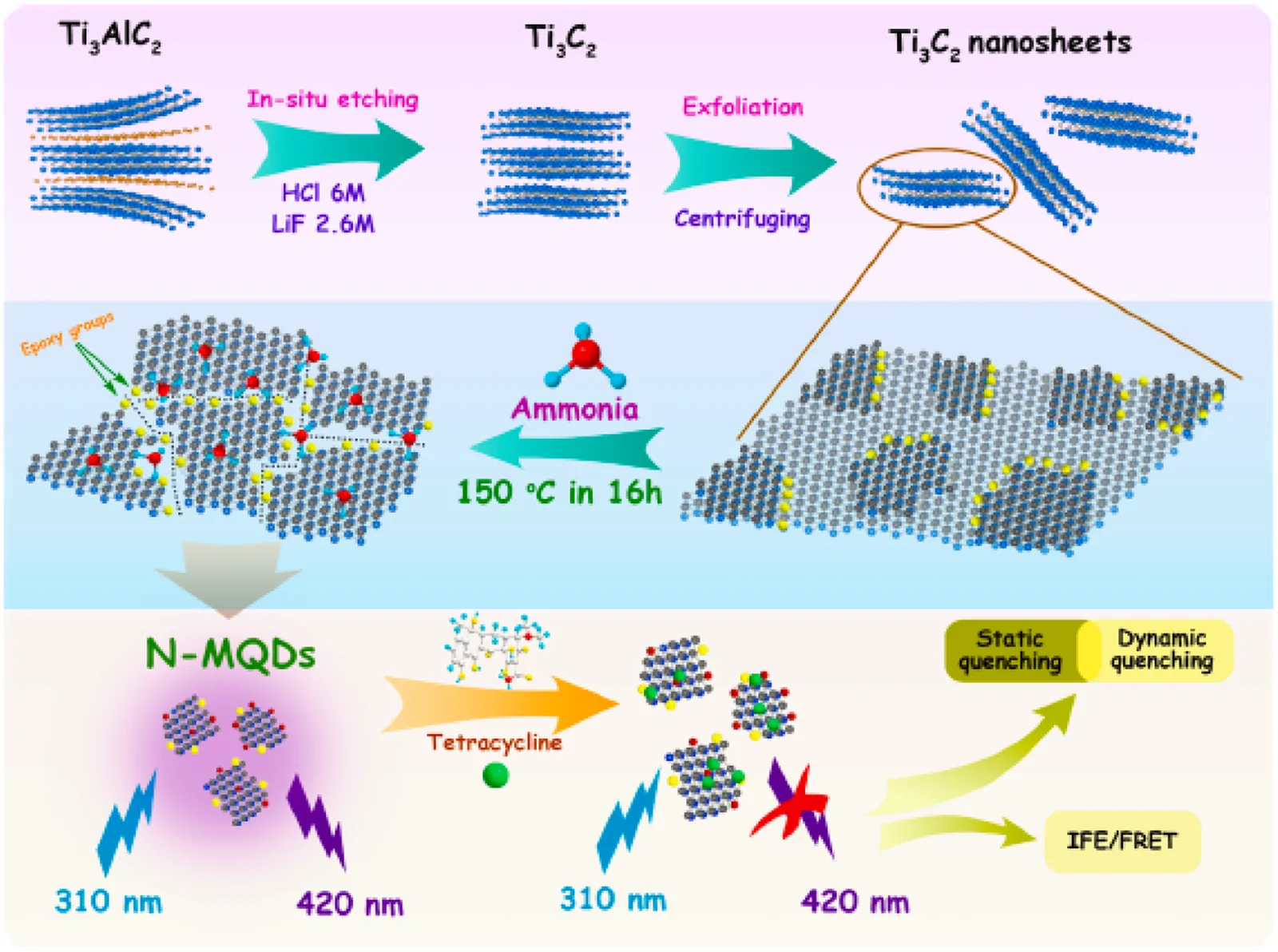
Synthesis of N-MXQDs and the sensing mechanism of TC. This figure has been adapted from ref. 117 with permission from Elsevier, copyright 2025.

Ti_3_C_2_ quantum dots produced from MXenes have lately become an intriguing sensing probe because of their superior optical characteristics. N-doped Ti_3_C_2_ quantum dots (N-MXQDs) were prepared by a hydrothermal process. The highest emission fluorescence of N-MXQDs is detected at 420 nm, and their fluorescence is excitation-dependent. The LOD of 24 nM was obtained by fitting the modified Stern–Volmer equation to the connection between the concentration of tetracycline (TC) and the quenching levels of N-MXQDs. The formation of a complex between N-MXQDs and TC has been quantitatively verified by the effective static quenching behaviour by N-MXQD sensors with superior selectivity for TC detection. The synthesis of N-MXQDs and the mechanism of TC sensing are shown in Fig. 1.^117^

Gram-positive *Bacillus anthracis* is regarded as a biological warfare agent, and is a major threat to living things. If the patient is not treated right away, inhaling 104 anthrax spores will result in death.^118–120^ The primary component of anthrax spores, 2,6-dipicolinic acid (DPA), makes up roughly 5–15% of the dry weight of *Bacillus anthracis* and is known to be a biomarker for bacillus spores.^121^ Consequently, it becomes crucial to detect DPA in order to implement *Bacillus anthracis* monitoring in a 48 hour period. The hydrothermal technique and easy acid oxidation were used to generate blue fluorescent MXQDs. The MXQDs exhibit good photostability and water solubility. With a linear range of 0–11 µM and a limit of detection of 0.26 nM, MXQDs were effectively utilized to construct a ratiometric fluorescence sensor for the detection of 2,6-dipicolinic acid (DPA) based on the absorbance energy-transfer effect (AETE). Additionally, a MXQD-based test paper having a low LOD of 52.4 nM was created for the optical detection of DPA.^122^

**Fig. 2:**
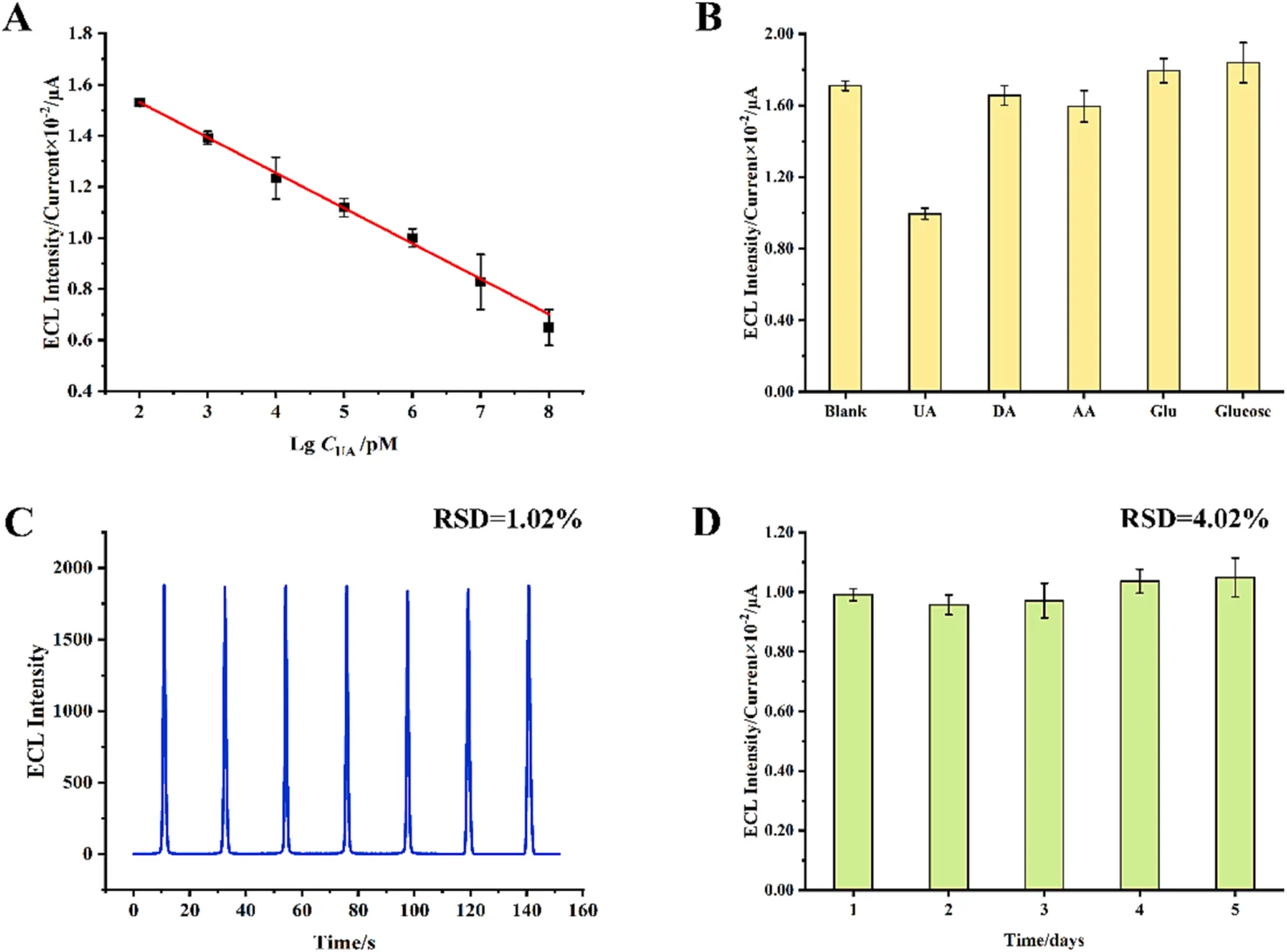
(A) Calibration curve of the prepared MIECLS for UA detection with the concentration range from 1 × 10^−10^ to 1 × 10^−4^ mol L^−1^. (B) Selectivity of the MIECLS towards UA and its interference. (C) ECL curves for the consecutive detection of 1 µmol per L UA by the MIECLS. (D) Stability of the MIECLS after storage and detection of 1 µmol per L UA. This figure has been adapted from ref. 125 with permission from Elsevier, copyright 2024.

Numerous illnesses, including hyperuricemia, gout, and several cardiovascular conditions, can result from having too much uric acid in the body, either because the body produces too much of it for excretion or because the system responsible for its excretion malfunctions.^123,124^ For the quick and sensitive detection of UA, an unlabeled ratiometric molecular imprinted electrochemiluminescence sensor has been developed using MXene@NaAsc nanocomposites, CdSe@ZnS quantum dots, and molecularly imprinted polymer composite-modified glassy carbon electrodes. Fig. 2 displays the performance of the molecular imprinted electrochemiluminescence sensor (MIECLS). With a limit of detection of 18.13 pmol L^−1^ (S/N = 3), the MIECLS can detect uric acid in the range of 1 × 10^−10^ to 1 × 10^−4^ mol L^−1^.^125^

In order to detect miRNA-155 with high sensitivity, Duan *et al.* devised a new impedimetric aptasensing technique based on a novel zero-dimensional (0D)/2D nanohybrid comprising Ti_3_C_2_T_*x*_ nanosheets decorated with FePc QDs (referred to as Ti_3_C_2_T_*x*_@FePcQDs). The cDNA-targeted miRNA-155 was suggested to be anchored by the Ti_3_C_2_T_*x*_@FePcQD nanohybrid acting as a nanocarrier. With a detection limit of 4.3 aM (S/N = 3) within the miRNA-155 concentration range of 0.01 fM to 10 pM, the Ti_3_C_2_T_*x*_@FePcQD-based aptasensor exhibits exceptional sensing properties for miRNA-155 detection. In order to ascertain if the created Ti_3_C_2_T_*x*_@FePcQD-based aptasensor could be used for long-term testing, its stability was assessed using EIS measurements. Finally, the electrochemical EIS response of the aptasensor was kept constant. Additionally, the signal maintained 103.7% of the initial signal, indicating that the current aptasensor is stable enough.^126^

### 3.2. MXQDs for cancer treatment

Early cancer diagnosis is the key to a successful recovery. X-ray, endoscopy, magnetic resonance imaging (MRI), computerized tomography (CT) scans, and biopsies have all been used extensively for decades to identify malignant tumors. However, these conventional diagnostic methods do have certain drawbacks, including high expenses, imprecision (false positive or negative), ignorance, needless therapies, and abuse of medical resources. The outcome of the diagnosis is also influenced by the tumor size, which increases the death rate. Therefore, a sophisticated, economical, sensitive, and biocompatible method for the early identification of tumor cells is desperately needed.^127^

T cells have a direct impact on bone cells, particularly osteoclasts, and are intimately associated with bone biology.^128^ MXQDs based on 0D Ti_3_C_2_ have been produced by Rafieerad *et al.* to improve tissue restoration following damage. In order to lessen inflammation and encourage tissue healing, MXQDs have demonstrated the immunomodulatory capacity to decrease the activation of particular T cells and encourage the growth of regulatory T cells.^129^

Traditional cancer therapeutic methods are insufficient despite their potential for invasiveness, high risk of tumor recurrence, and adverse effects. Because of their noninvasive characteristics and light trigger, photodynamic therapy (PDT) and photothermal therapy (PTT) present intriguing alternatives for the treatment of cancer.^130^

The MXQDs show promising result on tumor treatment utilizing photothermal therapy (PTT)/photoacoustic imaging (PAI).^43^ MXene-based PAI has overcome the limiting depth of imaging of traditional imaging techniques because of its low tissue attenuation coefficient, which makes it a potential imaging tool for deep tumor imaging. A technique utilizing small fluorescent V_2_CT_*x*_ QDs was proposed by Cao *et al.* They were discovered to have superior photothermal effects for cryogenic nuclear-targeted PTT in the NIR-II region.^131^ Additionally, they created V_2_CT_*x*_-TAT@Ex-RGD, a multifunctional thermal therapeutic platform modified with RGD. Furthermore, in the first and second biological windows of the NIR spectrum, Shao *et al.* discovered that both nitride-based MXenes and Ti_2_NT_*x*_ QDs have greater photothermal conversion efficiencies in NIR-I and NIR-II windows.^44^

An ultrasmall 2D V_2_C QD having outstanding photothermal effects for the NIR window was created by Cao *et al.*, which also has photoacoustic, fluorescence and MRI capabilities. V_2_C QDs were designed into a dual-target system for the nucleus and cell membrane. These QDs were encapsulated in endogenous exosomes after alteration with the *trans*-activator of transcription (TAT) peptides for targeting the nucleus (V_2_CTAT).^131^

**Fig. 3:**
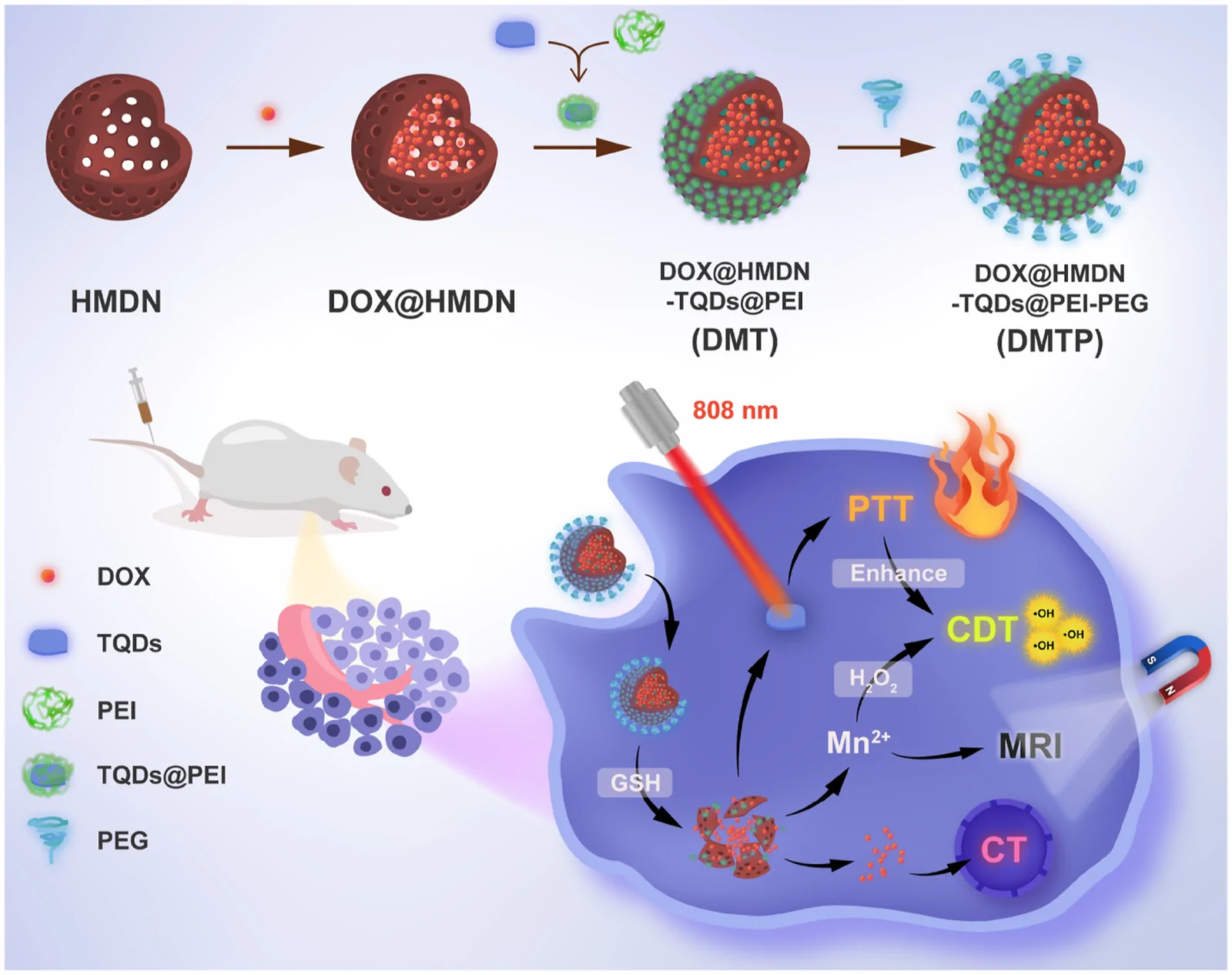
Synthesis of DOX@HMDN–TQDs@PEI–PEG (DMTP), CDT and MRI. This figure has been adapted from ref. 132 with permission from Elsevier, copyright 2024.

For cancer treatment, chemodynamic therapy (CDT) is a novel technique that eliminates cancer cells by catalyzing intracellular Fenton/Fenton-like reactions using low-valence transition metal ions, which produce hydroxyl radicals (˙OH). However, using CDT alone can make it difficult to achieve adequate therapeutic results. A hollow mesoporous MnO_2_ NP (HMDN)-based drug delivery system for cancer, DOX@HMDN–TQDs@PEI–PEG (DMTP), represented in Fig. 3, may improve cancer treatment by combining tumor magnetic resonance imaging (MRI) and synergistic therapeutics. Following the loading of the anticancer drug doxorubicin hydrochloride (DOX), the pores of HMDN were sealed using polyethyleneimine (PEI)-modified Ti_3_C_2_T_*x*_ QDs (TQDs). Polyethylene glycol (PEG) was then added to the finished system. HMDN produces Mn^2+^ that is utilized for both CDT and MRI, which depletes glutathione (GSH) in cancer cells, which is detrimental to CDT. Additionally, TQDs can be used to improve CDT by very effective photothermal therapy (PTT). Chemotherapy and DOX together can dramatically slow the growth of tumors.^132^

**Fig. 4:**
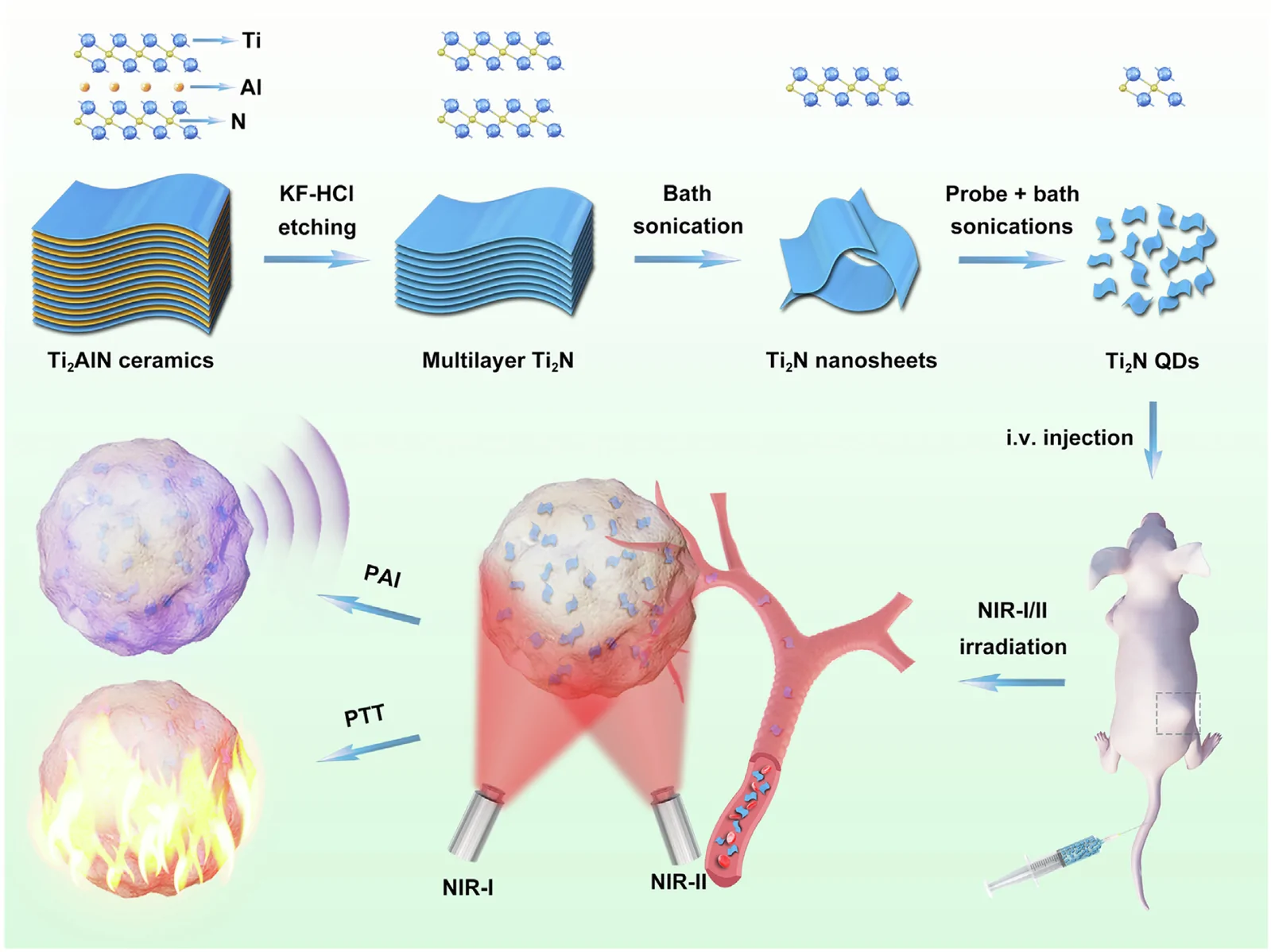
Ti_2_N QDs for PTT using PA imaging in both NIR-I and NIR-II biowindows. This figure has been adapted/reproduced from ref. 43 with permission from Elsevier, copyright 2020.

Using a top-down approach, Shao *et al.* synthesized Ti_2_N QDs. In both NIR-1 and NIR-II biowindows, Ti_2_N QDs demonstrate a remarkably high photothermal conversion efficiency under laser irradiation (NIR-I = 808 nm and NIR-II = 1064 nm). The Ti_2_N QDs exhibit outstanding photoacoustic (PA) effects, photothermal therapy (PTT) and biocompatibility according to *in vitro* and *in vivo* evaluations. Ti_2_N QDs as *in vivo* PTT agents in both NIR-I and NIR-II biowindows are displayed in Fig. 4. In both NIR-I (808 nm) and NIR-II (1064 nm) biowindows, Ti_2_N QDs exhibit strong photothermal stability under laser irradiation and exceptional photothermal properties with a very high photothermal conversion efficiency. Effective PA imaging-guided PTT in NIR-I/II biowindows is made possible by the slow accumulation of Ti_2_N QDs in tumors following tail vein delivery, which occurs without any discernible harm. Ti_2_N QDs with suitable degrading properties also exhibit a suitable excretion rate from the body *in vivo*, which is encouraging since it guarantees enough stability in circulation to carry out therapeutic functions before being easily expelled from the body.^43^

**Fig. 5:**
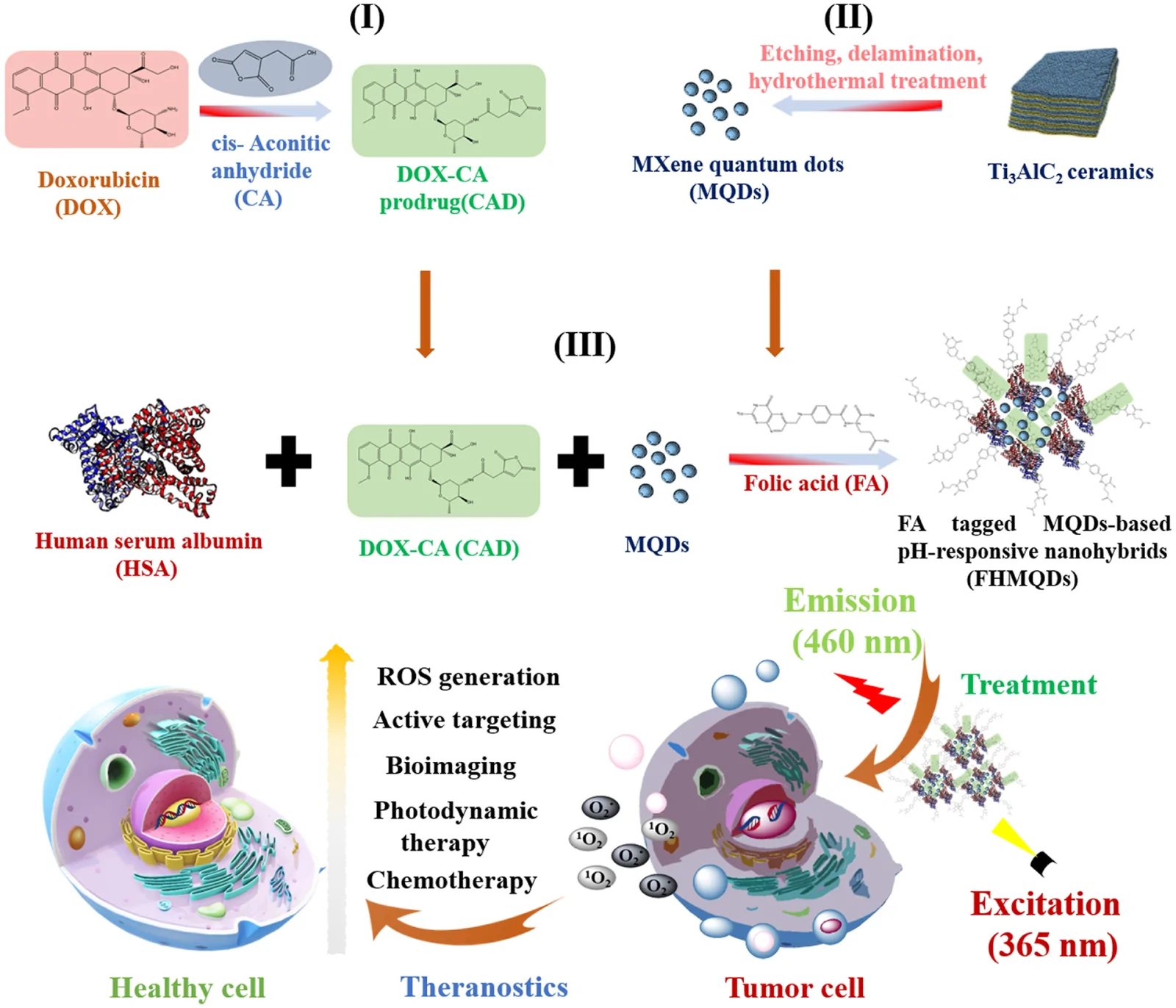
pH-responsive nanohybrid based on FA-tagged MXQD theranostics for breast cancer. This figure has been adapted from ref. 133 with permission from Elsevier, copyright 2025.

When encountered throughout a tumor microenvironment, Won *et al.* developed a prodrug system using pH-responsive MXQDs, which efficiently release doxorubicin (DOX), a chemotherapeutic drug. The produced MXQD-based system has a phototherapeutic impact for synergistic efficacy against cancer and is properly engineered for target selectivity. To create a safe and biocompatible platform, the synthesized MXQDs and *cis*-aconitic anhydride (CA), a pH-sensitive linker attached to DOX that is bonded to a human serum albumin (HSA) matrix, are shown in Fig. 5. Folic acid (FA), a ligand, adorns the surface of the manufactured system to provide target specificity. The efficacy of FHMXQDs in identifying and eliminating breast cancer cells was evaluated by comparing the *in vitro* cytotoxicity of various amounts of MXQDs, DOX, HMXQDs, and FHMXQDs with control utilizing the conventional MTT test in a continuous culture for 24 hours. DOX, HMXQDs, and FHMXQDs were shown to have respective IC_50_ values of 3.08 µg mL^−1^, 2.87 µg mL^−1^, and 2.24 µg mL^−1^. Furthermore, it was found that FHMXQDs exhibit great chemotherapeutic efficiency when exposed to no light, outperforming DOX and HMXQDs alone in the killing of cancer cells while having a comparatively low DOX concentration.^133^

Using advanced ultrasound technology and a hydrothermal reaction at atmospheric pressure, the Hu research group produced MXQDs. Additionally, MXQDs had the best photothermal conversion efficiency of comparable materials, reaching up to 62.5% at 808 nm. Without causing harm to the healthy tissue of mice, this led to considerable *in vitro* damage to cells and *in vivo* tumor regression. Remarkably, because MXQDs include Ti^3+^, they may react with H_2_O_2_ to form hydroxide groups (OH^−^) and hydroxyl radicals (˙OH), which can both reduce the amount of acid present in tumors and stop tumor growth. The potential use range of MXQDs is increased and their cost is decreased by using a straightforward and secure technique of synthesis. It may be used as a PTA to treat cancer.^134^

MXQDs and gold nanobones (Au NBs) were used as electrochemiluminescence (ECL) sensors. The greener method was used to prepare MXenes and MXQDs, for avoiding the negative impact of hydrofluoric acid on the environment. MiRNA-26a was detected using an ECL sensing device made of MXQD@Au NB heterostructures. The quenching effect of Au NBs can be lessened by MXQDs with certain electrochemical characteristics. However, Au NBs had superior charge transfer capabilities and an SPR effect, both of which enhanced ECL. In the ECL sensing technique, the bright signal of MXQDs may be effectively produced and greatly enhanced. However, in the heterostructure, the work functions of Au NBs and MXQDs with superior conductivity were somewhat similar. Triple-negative breast cancer patients had their serum levels of miRNA-26a measured by using the MXQD@Au NB-based ECL sensor.^135^ MXenes can be biocompatible and essentially safe at modest exposures, with minimal organ inflammation in mice after systemic administration and low acute mortality in zebrafish eggs. However, some animal models like chicken embryos have shown developmental harm at larger doses, and possible organ accumulation suggests further study. MXQD-mediated photothermal treatment for cancer suggested its potential for therapeutic use, which exhibits good biocompatibility and strongly inhibits the tumor with little damage to mice and without surface modification.^136^Table 5 shows the comparison of MXQDs with other materials used in cancer therapy. Won *et al.* developed FHMXQDs on MDA-MB 231 breast cancer cells, which showed excellent target specificity to breast cancer cells, which was reflected by its high cytotoxicity against cancer cells.^137,138^ Long-term toxicity necessitates thorough research on biodistribution, biodegradability, pharmacokinetics, and stability in intricate physiological settings. Therefore, clinical trials for MXQD-based cancer treatments have not yet been completed.^138^

**Table 5:** Comparison of MXQDs with other materials in cancer therapy

QDs	Drug	Target disease	Inference	Ref
AgQDs	Doxorubicin	Lung cancer	Significantly inhibit the migration of A549 cell	139
CdSeQDs	Methotrexate	Throat cancer	Higher cytotoxicity by drug conjugate than free MTX with 4 times more efficacy	140
CDs	Doxorubicin	Breast cancer	Drug release triggered at pH 7.2 was favourable for cancer cells	141
GQDs	Doxorubicin/curcumin	Colorectal and breast cancer	Higher drug accumulation at the tumour site than that of free drugs	142
V_2_C QDs	Ex with RGD	Cancer	Completely killing the tumor cells both *in vitro* and *in vivo*	143

### 3.3. MXQDs for drug delivery

BALB/c nude mice, free of pathogens, having HeLa tumor xenografts were used to study the anticancer effect of Ti_3_C_2_T_*x*_ MXQDs *in vivo*. The intratumoral therapy of MXQDs was studied at various concentrations in order to determine the therapeutic advantages. The body weights of the animals in the MXQD groups were comparable to those of the animals in the saline control group, suggesting that there was no discernible toxicity throughout the therapeutic therapy caused by the injection of MXQDs. Comparing Ti_3_C_2_T_*x*_ NMXQDs to the saline group, a noticeable reduction in tumor size was observed. Ti_3_C_2_T_*x*_ NMXQDs successfully inhibited tumor growth in HeLa cells following intratumor administration of the chemical, as demonstrated by the tumors of mice in different groups.^144^

### 3.4. MXQDs for bioimaging

Modern tumor diagnosis makes extensive use of bioimaging technology, which gives technicians clear and easy biological information through quick and simple approaches and serves as an imaging foundation for further treatment research.^145,146^ Conventional organic fluorescent materials exhibit cell-to-cell interactions, lack photostability, and are unable to follow cells. Because of their special small size and optical characteristics, quantum dots can infiltrate the nuclei and cell membranes for internal labeling, enabling more accurate medical diagnosis.^147^ Organic dyes with high QY are unsuitable for imaging because of their rapid photobleaching. Strong absorption and emission in the near-infrared (NIR-I and NIR-II) windows can be integrated into MXQDs, allowing for significantly deeper tissue imaging.^148–150^ In contrast to semiconductor QDs, which have high QY and greater photostability, their clinical translation is challenging because of their heavy metal content. MXQDs are resistant to photobleaching because of their strong chemical structures. MXQDs are now appropriate for imaging since they have a mild QY and are free of heavy metals.

Carbon dots (CDs) give similar quantum yields (10–40%) and have numerous advantages over MXQDs. However, MXQDs are perfect for imaging materials because CDs have no intrinsic photothermal activity. MRI contrast agents are better for whole-body scans because they provide deep tissue imaging. However, at shallow and moderate depths, MXQDs offer significantly higher spatial resolution.^151,152^ However, they do not have intrinsic fluorescence, enabling optical co-registration. Correlated microscopy is made possible by the dual optical/photoacoustic imaging capability of MXQDs. Compared to highly coated semiconductor QDs, their circulation period is lower; but, it is longer than that of free organic dyes.^153,154^ MXQDs perform similarly to carbon dots in terms of low cytotoxicity and outstanding biocompatibility. They break down into readily eliminated, non-toxic byproducts.^155,156^

Bioimaging is an important medical technology in diagnosis of cancer and its treatment. Imaging methods like MRI and CT scan are used for tissue analysis. Photothermal therapy (PTT) emerged as a promising method for cancer treatment. Photoacoustic (PA) imaging is a successful technique which allows for real-time analysis of organ vibrations during therapy. Near-infrared (NIR)-mediated PTT with chemotherapy has great ability for the treatment of cancer.^157^

Magnetic resonance imaging (MRI), supported PTT and chemotherapy offer detailed information about the size of the tumor, its location, and patient response to treatment. The convergence of imaging with therapies provide accurate and customized cancer treatment, which will improve the patient outcomes and survival.^158,159^

**Fig. 6:**
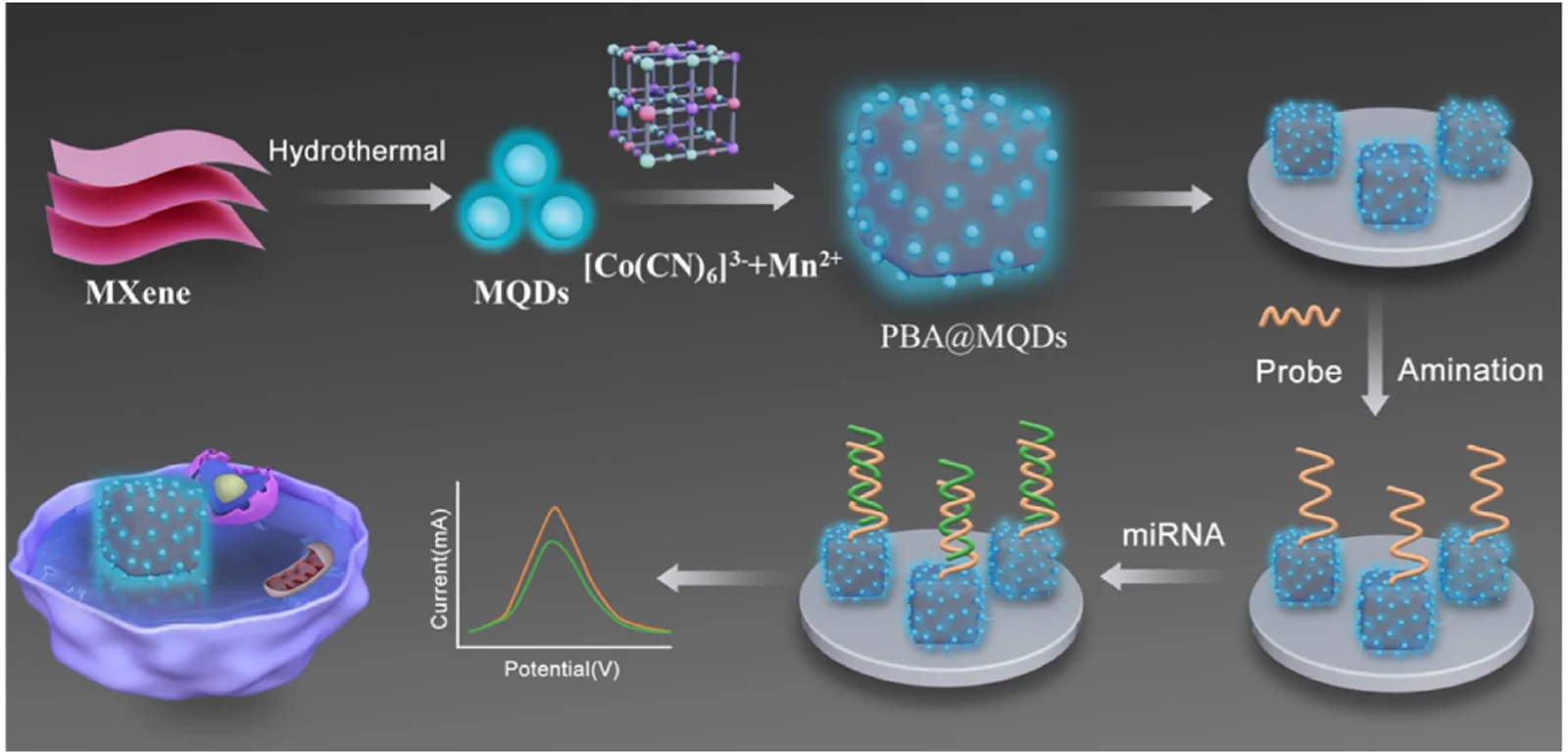
Co–Mn PBA@MXQDs NPs for the sensing and imaging of miRNA in living cells. This figure has been adapted from ref. 160 with permission from Elsevier, copyright 2025.

You *et al.* developed novel MXQD-coated bimetallic Prussian blue analogues (Co–Mn PBA@MXQDs) for sensing in *in vitro* and intracellular imaging of miRNA in living cells (Fig. 6). The 3D Co–Mn PBAs have the capacity to control the diffusion of metal clusters and ligand precursors that reduce the rate of reaction. This ensures that MXQDs are carriers of DNA probes. The resulting materials having miRNA recognition capabilities can show exceptional electrocatalytic and photoluminescence performance for miRNA identification.^160^

As MXQDs are small yet brilliant, they were used as bioimaging probes. The MXQDs can easily adsorb onto the cellular membrane and mark the cells due to their small size. The breast cancer cells (MCF-7) were incubated with an aqueous dispersion of MXQDs for two hours, and a confocal microscope was used to analyse the bioimaging performance of MXQDs.^161^

QDs show strong fluorescence when exposed to laser irradiation, and the cell viability was evaluated by a methylthiazolyldiphenyl-tetrazolium bromide (MTT) test. The QDs were incubated with MCF-7 cells for 4 and 24 hours. At the 200 and 400 µg mL^−1^ values, the viability was 90%. This suggests that these artificial MXQDs are extremely non-toxic. Additionally, using *X*-axis imaging, they verified that MXQDs had internalized into the cytoplasm of the cell. Glutathione was detected intracellularly using live-cell “turn-on” imaging and a Ti_3_C_2_ QDs/Fe^3+^ nanoprobe.^162^ When *N*-methylmaleimide is not preincubated, glutathione in cancer cells converts Fe^3+^ to Fe^2+^, restoring the fluorescence intensity of QDs and increasing the fluorescence of the cells. The generated sulfur and nitrogen-co doped Nb_2_C QDs were employed by Cai *et al.* for imaging Caco-2 cells.^163^

### 3.5. MXQDs for other applications

The device performance was enhanced by MXQDs due to their unique properties, enabling applications including self-powered wearable sensors for the detection of physiological signals like ECG, heart rate, and EMG and real-time health monitoring. According to current research, MXQDs are perfect for smart wearable devices because of their remarkable mechanical flexibility, electrical conductivity, and surface functionalization. The tunable optical and electronic properties of MXQDs allow their integration into smart multifunctional wearable platforms for health tracking, and electromagnetic interference shielding (EIS), which is a vital step toward the future generation of smart wearable systems.^164^

MXQDs are used as flexible strain and pressure sensors having high sensitivity and a broad range for strain detection. In wearable biosensors that track biomarkers like glucose and dopamine, they serve as conductive materials.^165^ Chandran *et al.* created N-doped MXQDs using a microwave-assisted method. The neurotransmitter norepinephrine is detected electrochemically and fluorescently using these spherical, bright, and incredibly sensitive N-MXQDs as dual probes.^166^

**Fig. 7:**
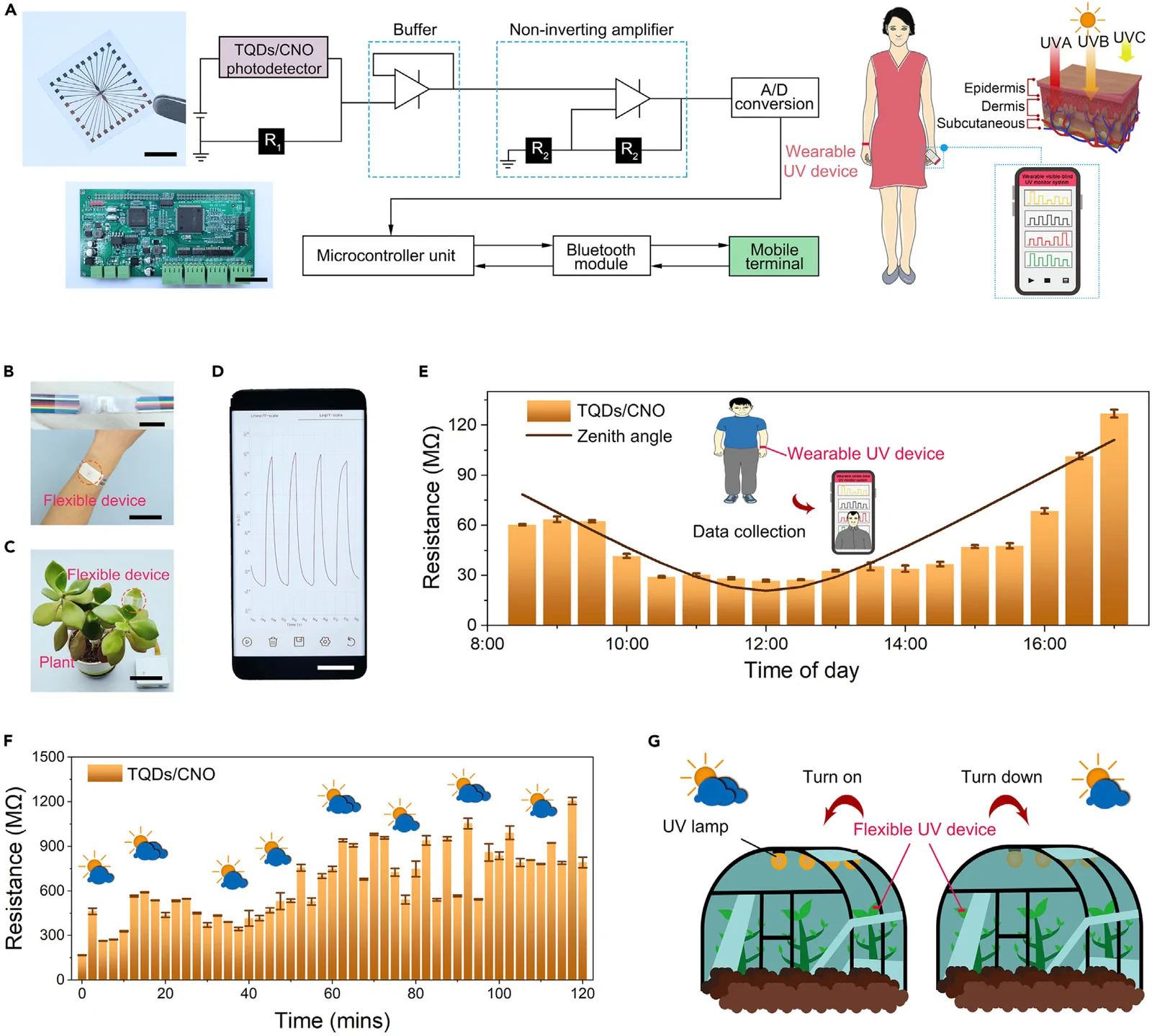
(A) Schematic of the sensing platform for UV-related wearable daily healthcare and control system. The photographs of the flexible TQD/CNO photodetector array (scale bar, 4 mm) and the printed circuit board (scale bar, 25 mm). (B) Optical images of flexible TQD/CNO photodetector on the wrist. Scale bar, 5 cm. Inset: zoomed-in image. Scale bar, 2 cm. (C) Optical images of flexible TQD/CNO photodetector on the plant. Scale bar, 5 cm. (D) Photograph of the mobile terminal. Scale bar, 2 cm. (E) The ambient UV radiation monitoring under sunny weather. The resistance of TQD/CNO photodetector (column) and zenith angle (curve) as a function of time. Values represent the mean and SD. *n* = 3. See also Fig. S13. (F) The ambient UV radiation monitoring under overcast weather. The cloud insets illustrate the evolution of cloud cover. Values represent the mean and SD. *n* = 3. (G) Schematic of wearable visible-blind UV moitoring system for controlling the UV irradiation. This figure has been adapted from ref. 167 with permission from Elsevier, copyright 2025.

Overexposure to UV radiation from sun damages the DNA of skin cells, causing skin aging, whereas in the case of plants, a little quantity of UV radiation is beneficial to regulate plant growth and improve the immune system. Thus, the Zheng research group developed a visible-blind UV-monitoring wearable device using a Ti_3_C_2_T_*x*_ QDs/Ca_2_Nb_3_O_10_ nanosheet photodetector. In order to replicate a system that measures UV radiation under different weather conditions, the TQD/CNO photodetector was coupled with a commercial data collector. The photodetector system array was connected with an artificial neural network (ANN) in wearable UV sensors for healthcare and user-alert systems. This may be improving the accuracy of information regarding the UV radiation in various weather conditions. The photodetector is connected to a data collector to replicate a wearable UV-monitoring system (Fig. 7A). To detect UV radiation from sunlight, the photodetector was positioned on the plant (Fig. 7C) and on the human wrist (Fig. 7B). The data collector transmitted the data to the cell phones using Bluetooth (Fig. 7D). A pre-warning signal was generated for UV exposure of the wearer to take precautions against overexposure. The sensing equipment also monitors ambient UV radiation in sunny conditions due to the flexibility of TQD/CNO photodetectors (Fig. 7E). With the increasing cloud cover, the resistance of the TQD/CNO photodetector also increases because of the negative correlation between the ambient UV radiation and the cloud cover, as seen in Fig. 7F. Fig. 7G shows a wearable visible-blind UV-monitoring system.^167^

To identify the stomach cancer marker miRNA-27a-3p, Li *et al.* created a new bimetallic MXene derivative QD (Mo_2_TiC_2_ QDs) as an electrochemiluminescence (ECL) emitter. It has been revealed that miRNA-27a-3p promotes GC cancer cells' invasion, migration, and proliferation (Guo and Li, 2020). Mo_2_TiC_2_ QDs exhibited excellent stability and spectacular ECL performances because of the extra carriers injected by inter-band excitation and the reduction in surface imperfections. It demonstrated high potential for identifying human gastric cancer using miRNA-27a-3p.^168^

Ascorbic acid (AA), an essential component in the human body for physiological activities, is critical for immunity and metabolism.^169,170^ A number of illnesses, including scurvy, Alzheimer's disease, and hypoimmunity, are strongly linked to aberrant AA levels.^171,172^ Determining AA in a sensitive and practical manner is therefore crucial. Acid phosphatase (ACP) is a common hydrolase that is mostly present in mammalian fluids and tissues, with the prostate having the highest activity.^173,174^ ACP is a crucial histological as well as serological biomarker that is frequently intimately linked to multiple myeloma, kidney disease, and prostate cancer.^175,176^ Ren *et al.* employed a solvothermal approach using amine molecules to synthesize N-doped Ti_3_C_2_ QDs (N-MXQDs) with bright blue fluorescence. Due to the good optical performance of N-MXQDs, it was paired with *o*-phenylenediamine (OPD) to develop a novel ratiometric fluorescence biosensor to monitor ascorbic acid (AA) as well as acid phosphatase (ACP). AA and ACP were detected with detection limits as low as 0.82 µM and 0.02 U L^−1^. Furthermore, this fluorescence sensor's response time for determining AA and ACP was less than one and a half hours.^177^

Immuno-engineered tantalum carbide (Ta_4_C_3_T_*x*_) MXene quantum dots: design, production, characterization, and use for both *in vitro* and *in vivo* immunomodulatory applications. These Ta_4_C_3_T_*x*_ MXQDs were logically created for use in biomedicine using a customized hydrothermal, etching, and exfoliation procedure. Excellent biocompatibility with human cells was facilitated by the large amounts of MXene surface functional groups and surface tantalum oxides (TaO_2_ and Ta_2_O_5_) present in the as-synthesised MXQDs. In particular, human endothelial cells (ECs) grown in high concentrations of Ta_4_C_3_T_*x*_ MXQDs did not experience cytotoxicity or oxidative stress. Additionally, these MXQDs were absorbed into ECs on their own, and by controlling T-cell activation, mechanistically helped to lower the immunogenicity of these cells. Lastly, Ta_4_C_3_T_*x*_ MXQDs administered intravenously decreased both immune cell infiltration and structure degeneration within transplanted tissues in an *in vivo* model of transplanted organ rejection.^178^

In order to detect influenza A virus (FluA) and severe acute respiratory syndrome coronavirus 2 (SARSCoV-2) concurrently, Liu *et al.* created a novel colorimetric and fluorescent dual-functional two-channel immunochromatographic assay (ICA) biosensor. The Ti_3_C_2_-QD-ICA biosensor has a short testing time (20 min), strong repeatability, specificity, and accuracy, and can concurrently detect 1 ng mL^−1^ or 2.4 pg per mL FluA and 1 ng mL^−1^ or 6.2 pg per mL SARS-CoV-2 *via* its colorimetric or fluorescence signals, respectively. Ti_3_C_2_-QD-ICA was used to assess throat swab samples that had been spiked with different concentrations of H1N1 and SARS-CoV-2 virions in order to further investigate the biosensor's effectiveness in practical applications. According to Table 6, the Ti_3_C_2_-QD-ICA biosensor's CV for H1N1 and SARS-CoV-2 viral specimens ranged from 5.11% to 8.39%, with a recovery of 84.70% to 114.00%. These results showed that the biosensor could accurately determine the relative quantity of the two target viruses. As confirmed by the quantitative polymerase chain reaction (qPCR), nasopharyngeal swabs were then obtained from participants, containing 10 positive H1N1, 10 positive SARS-CoV-2, and 10 negative cases.^179^

**Table 6:** Ti_3_C_2_-QD-ICA for the recovery of H1N1 and SARS-CoV-2

Virus	Ti_3_C_2_-QD-ICA in copies per mL	Detection in copies per mL	Recovery (%)	CV (%)
H1N1	10^6^	8.47 × 10^5^	84.70	5.76
	10^3^	8.56 × 10^2^	85.60	7.86
SARS-CoV-2	10^5^	1.14 × 10^5^	114.00	8.39
	10^4^	9.65 × 10^3^	96.50	5.11

Carbon-doped MXene quantum dots (MXQDs) were prepared using MXenes, citric acid (CA) and ethylene diamine. MILPdMXNQDs were generated by incorporating palladium nanoparticles into MNQDs and placing them on an Fe-based metal–organic framework. l-ascorbic acid (AA) concentrations in a phosphate buffer solution ranged from 10 to 100 nM, and were measured with modified glassy carbon and screen-printed electrodes incorporating MIL-PdMNQDs. This sensor demonstrated selective detection in fake sweat, detecting the concentrations of AA between 100 and 1000 µM.^180^

Researchers are very interested in the nanomaterialization of traditional Chinese medicine (TCM). One type of TCM that has significant uses in preventing wound infections is sanguinarine (SAN), which has strong antibacterial qualities. Furthermore, bacterial resistance can be overcome by combining photothermal therapy with chemotherapy, which increases the effectiveness of bactericidal and wound-healing treatments. In order to achieve pH/NIR-controlled drug release and photothermal therapy/chemotherapy synergistic antibacterial and wound healing, Zhang *et al.* loaded SAN onto a zwitterion-modified MXene quantum dot nanocarrier (SAN@AHEP@Ta_4_C_3_) to create an antibacterial agent. SAN@AHEP@Ta_4_C_3_ has a particle size of around 120 nm and is stable and soluble in water.^181,182^

**Fig. 8:**
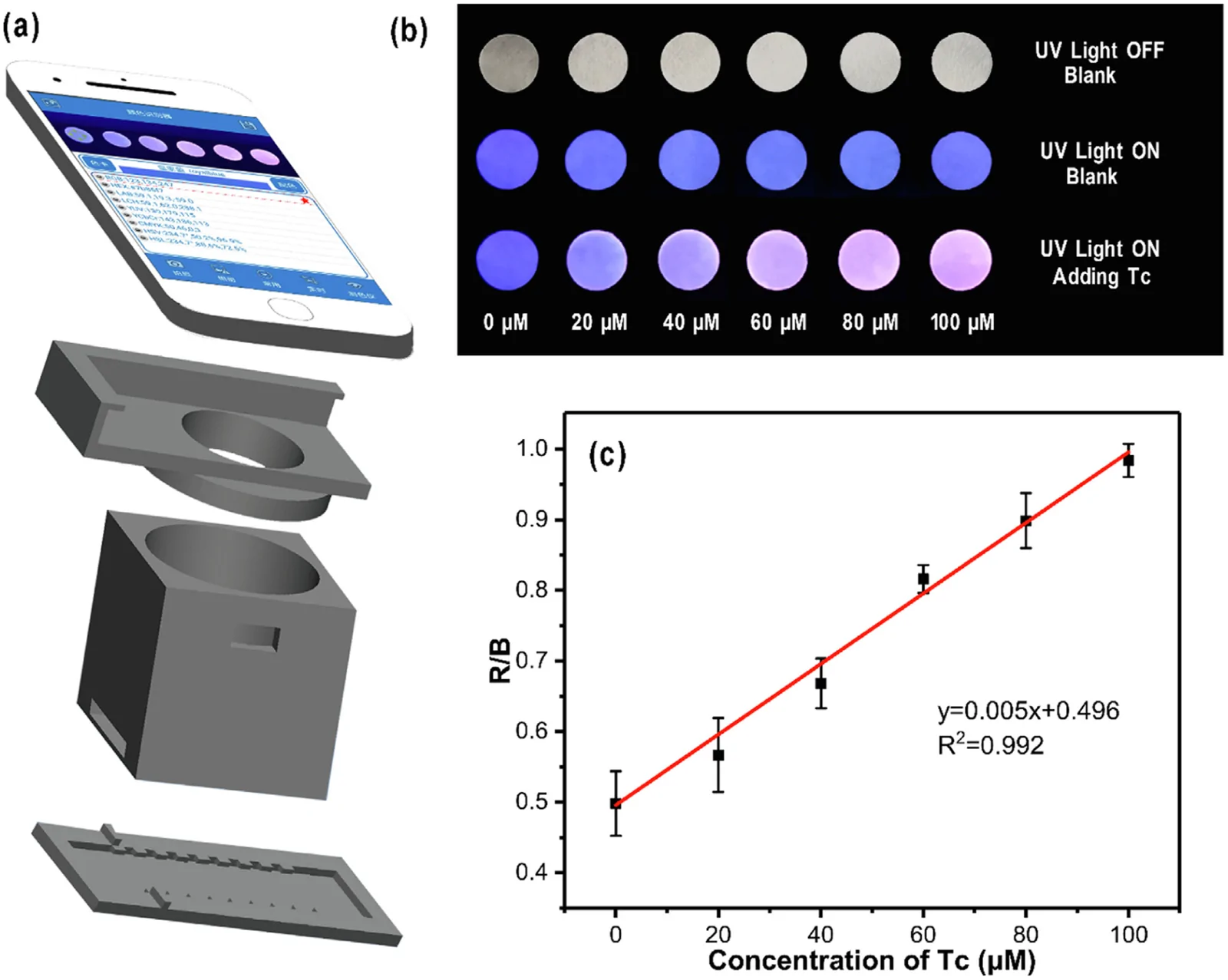
Tc detection: (a) smart device, (b) colorimetric card for Tc detection and (c) relationship between R/B and Tc concentrations. This figure has been adapted from ref. 183 with permission from Elsevier, copyright 2022.

Tetracycline (Tc), a broad-spectrum antibiotic, is widely used for the prevention as well as treatment of diseases in animals. However, the overuse of antibiotics causes environmental contamination due to its excretion in animal waste and antibiotic buildup in animal-derived foods.^182^ To measure Tc rapidly and precisely, a ratiometric fluorescence (RFL) method based on the antenna effect (AE) and Foster resonance energy transfer (FRET) was developed. Utilizing the synergistic effects of the FRET from Ti_3_C_2_ QDs to Eu^3+^ ions and the antenna effect (AE) between Tc and Eu^3+^ ions, europium ions and Ti_3_C_2_ QDs worked together to create an RFL for Tc detection. Furthermore, we proposed a ratiometric FL platform for Tc detection having a low detection limit (48.79 nM). It has a detection limit of 48.79 nM, and is effective for detecting Tc in soil and milk. Fu *et al.* developed a gadget utilizing a smartphone for the detection of Tc (Fig. 8a). The image was detected by photographing the test paper, and the RGB color shift was identified using the smartphone's app. When the QDs/Eu^3+^ were sprayed on filter paper, as shown in Fig. 8b, the paper could not emit any light after switching off the UV lamp. As shown in Fig. 8c, the relation between the R/B ratio and the Tc concentration in the range of 0–100 µM demonstrated a linear calibration curve. This confirmed that the portable device was successfully prepared for real-time, on-site analysis for sensitive detection.^183^

## 4. Clinical translation: limitations and challenges

With regard to their distinct characteristics, MXQDs have become popular choices for medical applications. However, in order to efficiently use the advantages of MXQDs, a number of limitations and difficulties must be resolved.

There are still a number of obstacles which have to be resolved about their potential biomedical uses, particularly with regard to their selectivity, toxicity, and biocompatibility. In order to investigate their long-term toxicity, histopathology, biodistribution/biodegradability, and photoluminescence characteristics, clinical trials and comprehensive *in vivo*/*in vitro* research are also expected. Another significant issue is the toxicological impact of MXQDs, which requires further investigation into both *in vitro* and *in vivo* aspects to ensure their safe use.

Research on the biocompatibility of MXQDs is still minimal, and it should focus on thorough toxicity assessment and reduction. The cytotoxicity, uptake behavior, and *in vitro* and *in vivo* toxicity of MXQDs should all be studied. Because MXQDs have the ability to accumulate in human bodies, their physiological effects must be properly explored and understood. The relationship between MXQDs and human physiological systems has not been fully investigated yet.

MXenes continue to degrade, despite developments that improve their stability in biological settings.^184^ As MXenes are prepared from early transition metals, they accumulate in the liver, and small quantities are also found in the spleen, heart, lungs, and kidneys, according to biodistribution studies. As a result of increased excretion through urine and feces, their concentrations gradually decline.^185^ Variable forms of altered MXenes have quite variable half-lives in plasma. For instance, GdW10@MXene has a short half-life of 49.5 minutes compared to Nb_2_C–PVP, which has a half-life of 3.8 hours. MXenes are mostly biodegraded in the liver and gastrointestinal tract, which is essential to their metabolic removal.^186,187^ MXenes accumulate in the spleen which acts as a clearance organ, where the particle size larger than ∼100–200 nm are excluded, influencing biodistribution and also clearance.^188^ According to research, MXenes produce more reactive oxygen species (ROS) than their MAX phase predecessors. Oxidative stress is frequently linked to the toxicity of 2D materials. Notably, this increased toxicity was directly linked to physicochemical interactions with cellular membranes and also ROS production, highlighting the complex nature of MXene-induced cytotoxicity.^189^ The material structure, dosage, length of exposure, and cell phenotype all influence the toxicity of MXenes, which is highly context-dependent. These results highlight the need for future toxicity studies to incorporate metabolic, oxidative, and membrane-interaction analyses in order to promote the safe design and biological translation of MXene-based products.^190^

Biocompatibility and long-term biosafety is another important challenge. Current *in vivo* studies reveal that organ-specific accumulation, sublethal metabolic effects and oxidative stress are not detected by standard assays, despite early *in vitro* research suggesting that polymer-wrapped MXQDs demonstrate less acute cytotoxicity.

From a regulatory point of view, the clinical implementation of multifunctional nanomedicines is hindered by the absence of defined evaluation frameworks. The multifunctional properties of MXQDs are not fully taken into consideration by present regulations, which demand clear evidence of efficacy, safety and reproducible manufacture. Clinical translation requires standardized procedures for functional performance standards, batch consistency, and physicochemical analysis.^191^

To address these obstacles, interdisciplinary cooperation between chemists, pharmacologists, materials scientists, and regulatory specialists is essential. In order to advance MXQDs from lab-scale research to safe and efficient clinical treatments, it is essential to address scalability, thorough safety profiling, nano-bio interactions, and regulatory readiness.^192^ Although preclinical research has demonstrated the potential of MXenes, thorough *in vivo* and clinical trials are required to assess their long-term effects, safety, and efficacy. Successful translation into clinical settings will depend on understanding how they interact with the immune system and reducing any undesirable reactions.^193^

In addition, the primary problem that needs to be resolved for upcoming broad pharmacological, biological and clinical applications is the production of less hazardous QDs. Through a process called biosynthesis, biological systems like bacteria, fungi, yeasts, algae, and plants can produce less harmful QDs. It is also possible to create less hazardous water-soluble QDs for use in clinical applications. Research on QDs is still in its initial stages, with few clinical trials, yet significant preclinical developments.

## 5. Conclusion and future perspectives

MXenes and the resulting MXQDs have become an emerging field and a hotspot for 2D materials due to their potential for sophisticated applications. MXQDs are perfect for biological applications because of their huge specific surface areas, exceptional photoelectric qualities, and distinctive 2D structures. In the near future, more investigation on these quantum dots may result in better cancer treatments. Despite the significant advancements in tumor detection, diagnosis, and therapy, QDs continue to encounter a number of obstacles pertaining to surface functionalization, biocompatibility, manufacturing procedures, and potential environmental risks.

Large-scale MXQD production is linked to environmental risks because of organic solvents or hazardous heavy metals. Chemical alterations, caused by biocompatible polymers, can enhance MXQDs' surface characteristics and increase their suitability for biomedical applications. However, careful oversight is required while synthesizing MXQDs for drug carriers. Regulating MXQDs for high loadings of drugs is thus an essential component for cancer treatment.

Recent advances in intelligent MXQD-based nanosystems have shown their promising therapeutic potential for effective and immunomodulatory cancer therapy. MXQDs are becoming more and more popular because of their exceptional optical-electrical and physicochemical characteristics. The rapid advancements in exfoliation techniques have facilitated the production of MXQDs with a range of special qualities, including high selectivity to target analytes, low toxicity, good biocompatibility, and excellent PL properties. These properties have laid the groundwork for the wide-ranging applications of MXQDs in sensing, bioimaging, cancer treatment, optoelectronics, catalysis, and battery production, among other fields. Notwithstanding these outstanding accomplishments, this intriguing but young field still faces numerous basic and technological shortcomings and difficulties.

With enormous potential for next-generation smart wearable and high-performance optoelectronics, MXQDs constitute a quickly expanding field in nanomaterial science. The use of MXQD-based sensors for wearable and portable point-of-care (PoC) analysis requires further research. Wearable sensors, along with artificial intelligence (AI), internet of things (IoT) and biosensing technologies, have the potential to provide individualized health tracking through continuous and non-invasive diagnostics. The goal of this thorough analysis is to be a useful tool for practitioners and researchers in the fields of biosensing, environmental monitoring, healthcare and materials science.

## Conflicts of interest

There are no conflicts to declare.

## Data Availability

No primary research results have been included, and no new data were generated or analysed as part of this review.

## References

[cit1] Chakroborty S., Nath N., Sahoo S., Singh B. P., Bal T., Tiwari K., Hailu Y. K., Singh S., Kumar P., Chakraborty C. (2025). Nanoscale Adv..

[cit2] Chakroborty S., Nath N., Bal T., Panda A. R., Hailu Y. K., Soren S., Tiwari K., Khan R., Kumari K., Chakraborty C. (2025). Bionanoscience.

[cit3] Nath N. , Chakroborty S. and Moharana S., in Carbon Nanotube-Polymer Nanocomposites: Synthesis, Properties, and Applications, Springer Nature Singapore, Singapore, 2024, pp. 311–330

[cit4] Chakroborty S., Nath N., Mahal A., Barik A., Panda A. R., Bal T., Obaidullah A. J., Elawady A. (2024). J. Mol. Liq..

[cit5] Yang G., Liu F., Zhao J., Fu L., Gu Y., Qu L., Zhu C., Zhu J. J., Lin Y. (2023). Coord. Chem. Rev..

[cit6] Huang K., Li Z., Lin J., Han G., Huang P. (2018). Chem. Soc. Rev..

[cit7] Jia L., Zhou S., Ahmed A., Yang Z., Liu S., Wang H., Li F., Zhang M., Zhang Y., Sun L. (2023). Chem. Eng. J..

[cit8] Huang H., Dong C., Feng W., Wang Y., Huang B., Chen Y. (2022). Adv. Drug Delivery Rev..

[cit9] Zarco-Fernández S., Coto-García A. M., Munoz-Olivas R., Sanz-Landaluze J., Rainieri S., Camara C. (2016). Chemosphere.

[cit10] Wiecinski P. N., Metz K. M., King Heiden T. C., Louis K. M., Mangham A. N., Hamers R. J., Heideman W., Peterson R. E., Pedersen J. A. (2013). Environ. Sci. Technol..

[cit11] Takagahara T., Takeda K. (1992). Phys. Rev. B:Condens. Matter Mater. Phys..

[cit12] Karakoti A. S., Shukla R., Shanker R., Singh S. (2015). Adv. Colloid Interface Sci..

[cit13] Ding N., Zhou F., Li G., Shen H., Bai L., Su J. (2024). Mater. Today Bio.

[cit14] Ekimov A. I., Onushchenko A. A. (2023). JETP Lett..

[cit15] Rocha T. L., Mestre N. C., Sabóia-Morais S. M. T., Bebianno M. J. (2017). Environ. Int..

[cit16] Ekimov A. I., Onushchenko A. A. (1981). JETP Lett..

[cit17] Pal A., Arshad F., Sk M. P. (2020). Adv. Colloid Interface Sci..

[cit18] Jang E., Jang H. (2023). Chem. Rev..

[cit19] Agarwal K., Rai H., Mondal S. (2023). Mater. Res. Express.

[cit20] Abd Rani U., Ng L. Y., Ng C. Y., Mahmoudi E. (2020). Adv. Colloid Interface Sci..

[cit21] Sun P., Xing Z., Li Z., Zhou W. (2023). Chem. Eng. J..

[cit22] Li G., Liu Z., Gao W., Tang B. (2023). Coord. Chem. Rev..

[cit23] Naguib M., Barsoum M. W., Gogotsi Y. (2021). Adv. Mater..

[cit24] Vahid Mohammadi A., Rosen J., Gogotsi Y. (2021). Science.

[cit25] Gogotsi Y., Huang Q. (2021). ACS Nano.

[cit26] Coleman J. N., Lotya M., O'Neill A., Bergin S. D., King P. J., Khan U., Young K., Gaucher A., De S., Smith R. J., Shvets I. V. (2011). Science.

[cit27] Kailasa S. K., Joshi D. J., Koduru J. R., Malek N. I. (2021). J. Mol. Liq..

[cit28] Naguib M., Kurtoglu M., Presser V., Lu J., Niu M., Heon J., Hultman L., Gogotsi Y., Barsoum M. W. (2011). Adv. Mater..

[cit29] Peera S. G., Liu C., Sahu A. K., Selvaraj M., Rao M. C., Lee T. G., Koutavarapu R., Shim J., Singh L. (2021). Adv. Mater. Interfaces.

[cit30] Rhouati A., Berkani M., Vasseghian Y., Golzadeh N. (2022). Chemosphere.

[cit31] Sinha A., Zhao H., Huang Y., Lu X., Chen J., Jain R. (2018). TrAC, Trends Anal. Chem..

[cit32] Sun T., Tang M., Shi Y., Li B. (2022). Chem. Rec..

[cit33] Xu Q., Cai W., Li W., Sreeprasad T. S., He Z., Ong W. J., Li N. (2018). Mater. Today Energy.

[cit34] Jin Z., Liu C., Liu Z., Han J., Fang Y., Han Y., Niu Y., Wu Y., Sun C., Xu Y. (2020). Adv. Energy Mater..

[cit35] Iravani S., Varma R. S. (2022). Nanomaterials.

[cit36] Abdelsalam H., Zhang Q. F. (2022). Adv. Phys.:X.

[cit37] Shao B., Liu Z., Zeng G., Wang H., Liang Q., He Q., Cheng M., Zhou C., Jiang L., Song B. (2020). J. Mater. Chem. A.

[cit38] Rambabu D., Bhattacharyya S., Singh T., ML C., Maji T. K. (2020). Inorg. Chem..

[cit39] Rafieerad A., Yan W., Sequiera G. L., Sareen N., Abu-El-Rub E., Moudgil M., Dhingra S. (2019). Adv. Healthcare Mater..

[cit40] Zhu Y., Feng L., Zhao R., Liu B., Yang P. (2024). ACS Appl. Nano Mater..

[cit41] Rafieerad A., Yan W., Alagarsamy K. N., Srivastava A., Sareen N., Arora R. C., Dhingra S. (2021). Adv. Funct. Mater..

[cit42] Hu B., Chen J., Gao Z., Chen L., Cao T., Li H., Yu Q., Wang C., Gan Z. (2024). ACS Appl. Bio Mater..

[cit43] Shao J., Zhang J., Jiang C., Lin J., Huang P. (2020). Chem. Eng. J..

[cit44] Xue Q., Zhang H., Zhu M., Pei Z., Li H., Wang Z., Huang Y., Huang Y., Deng Q., Zhou J., Du S. (2017). Adv. Mater..

[cit45] Dai W., Dong H., Zhang X. (2018). Materials.

[cit46] Cao Y., Wu T., Zhang K., Meng X., Dai W., Wang D., Dong H., Zhang X. (2019). ACS Nano.

[cit47] Rafieerad A., Yan W., Amiri A., Dhingra S. (2020). Mater. Des..

[cit48] Vahid Mohammadi A., Rosen J., Gogotsi Y. (2021). Science.

[cit49] Naguib M. , Kurtoglu M., Presser V., Lu J., Niu J., Heon M., Hultman L., Gogotsi Y. and Barsoum M. W., in MXenes, Jenny Stanford Publishing, 2023, pp. 15–29

[cit50] Anasori B. , Lukatskaya M. R. and Gogotsi Y., in MXenes, Jenny Stanford Publishing, 2023, pp. 677–722

[cit51] Shekhirev M., Shuck C. E., Sarycheva A., Gogotsi Y. (2021). Prog. Mater. Sci..

[cit52] Vénosová B., Karlický F. (2025). Nanoscale.

[cit53] Cipriano L. A., Di Liberto G., Tosoni S., Pacchioni G. (2020). Nanoscale.

[cit54] Fan T., Yan L., He S., Hong Q., Ai F., He S., Ji T., Hu X., Ha E., Zhang B., Li Z. (2022). Chem. Soc. Rev..

[cit55] Lv Z., Ma W., Wang M., Dang J., Jian K., Liu D., Huang D. (2021). Adv. Funct. Mater..

[cit56] Sherryna A., Tahir M. (2022). Chem. Eng. J..

[cit57] Wang X., Wang C., Ci S., Ma Y., Liu T., Gao L., Qian P., Ji C., Su Y. (2020). J. Mater. Chem. A.

[cit58] Xu Y., Wang X., Zhang W. L., Lv F., Guo S. (2018). Chem. Soc. Rev..

[cit59] Alijani H., Rezk A. R., Khosravi Farsani M. M., Ahmed H., Halim J., Reineck P., Murdoch B. J., El-Ghazaly A., Rosen J., Yeo L. Y. (2021). ACS Nano.

[cit60] Bartkowski M., Zhou Y., Nabil Amin Mustafa M., Eustace A., Giordani S. (2024). Chem.–Eur. J..

[cit61] Bruno F., Sciortino A., Buscarino G., Soriano M., Rios A., Cannas M., Agnello S. (2021). Nanomaterials.

[cit62] Khayal A., Dawane V., Amin M., Tirth V., Yadav V., Algahtani A., Jeon B. (2021). Polymers.

[cit63] Babu A. M., Rajeev R., Thadathil D. A., Varghese A., Hegde G. (2022). J. Nanostruct. Chem..

[cit64] Cai G., Yu Z., Tong P., Tang D. (2019). Nanoscale.

[cit65] Neupane G. P., Wang B., Tebyetekerwa M., Nguyen H. T., Taheri M., Liu B., Nauman M., Basnet R. (2021). Small.

[cit66] Huang D., Wu Y., Ai F., Zhou X., Zhu G. (2021). Sens. Actuators, B.

[cit67] Alhabeb M., Maleski K., Anasori B., Lelyukh P., Clark L., Sin S., Gogotsi Y. (2017). Chem. Mater..

[cit68] Zhang Q., Sun Y., Liu M., Liu Y. (2020). Nanoscale.

[cit69] Bagyalakshmi S., Thilakavathi G., Sivakami A., Sukumar T., Gowri S. (2026). Mater. Sci. Semicond. Process..

[cit70] Yu X., Cai X., Cui H., Lee S. W., Yu X. F., Liu B. (2017). Nanoscale.

[cit71] Han M., Yang J., Jiang J., Jing R., Ren S., Yan C. (2021). J. Colloid Interface Sci..

[cit72] Iravani S., Varma R. (2022). Nanomaterials.

[cit73] Sobhanan J., Rival J., Anas A., Shibu E., Takano Y., Biju V. (2023). Adv. Drug Delivery Rev..

[cit74] Guan Q., Ma J., Yang W., Zhang R., Zhang X., Dong X., Fan Y., Cai L., Cao Y., Zhang Y., Li N. (2019). Nanoscale.

[cit75] Ai F., Fu C., Cheng G., Zhang H., Feng Y., Yan X., Zheng X. (2021). ACS Appl. Nano Mater..

[cit76] Zhao L., Wang Z., Li Y., Wang S., Wang L., Qi Z., Ge Q., Liu X., Zhang J. Z. (2021). J. Mater. Sci. Technol..

[cit77] Devi S., Kumar M., Tiwari A., Tiwari V., Kaushik D., Verma R., Bhatt S., Sahoo B. M., Bhattacharya T., Alshehri S., Ghoneim M. M. (2022). Front. Mater..

[cit78] Sun J., Shengping Zhang B. S., Alomar M., Alqarni A. S., Najla Alotaibi M. S., Badriah Alshahrani M. S., Alghamdi A. A., Kou Z., Shen W., Chen Y., Zhang J. (2023). Chem. Rec..

[cit79] Cheng H., Ding L. X., Chen G. F., Zhang L., Xue J., Wang H. (2018). Adv. Mater..

[cit80] Kruger D. D., García H., Primo A. (2024). Adv. Sci..

[cit81] Thörnberg J., Halim J., Lu J., Meshkian R., Palisaitis J., Hultman L., Persson P. O., Rosen J. (2019). Nanoscale.

[cit82] Downes M., Shuck C. E., McBride B., Busa J., Gogotsi Y. (2024). Nat. Protoc..

[cit83] Zhan X., Si C., Zhou J., Sun Z. (2020). Nanoscale Horiz..

[cit84] Singh R., Kumar S., Bera S., Bhunia S. K. (2023). ACS Appl. Nano Mater..

[cit85] Das S., Mondal S., Ghosh D. (2024). Front. Bioeng. Biotechnol..

[cit86] Shuck C. E., Ventura-Martinez K., Goad A., Uzun S., Shekhirev M., Gogotsi Y. (2021). ACS Chem. Health Saf..

[cit87] Amendola V., Polizzi S., Meneghetti M. (2006). J. Phys. Chem. B.

[cit88] Rahman A., Guisbiers G. (2024). Metals.

[cit89] Ramírez-Grau R., Cabrero-Antonino M., García H., Primo A. (2024). Appl. Catal., B.

[cit90] Ramírez R., Melillo A., Osella S., Asiri A. M., Garcia H., Primo A. (2023). Small Methods.

[cit91] Huang D., Xie Y., Lu D., Wang Z., Wang J., Yu H., Zhang H. (2019). Adv. Mater..

[cit92] Shivappanayaka A., Elamkulavan H. J., Reddy V. S., Keloth C. (2025). Nanoscale Adv..

[cit93] Fan Y., Li L., Zhang Y., Zhang X., Geng D., Hu W. (2022). Adv. Funct. Mater..

[cit94] Manawi Y. M., Samara A., Al-Ansari T., Atieh M. A. (2018). Materials.

[cit95] Wang Y., Li C., Han X., Liu D., Zhao H., Li Z., Xu P., Du Y. (2018). ACS Appl. Nano Mater..

[cit96] Liu M., Bai Y., He Y., Zhou J., Ge Y., Zhou J., Song G. (2021). Microchim. Acta.

[cit97] Khazaei M., Arai M., Sasaki T., Chung C. Y., Venkataramanan N. S., Estili M., Sakka Y., Kawazoe Y. (2013). Adv. Funct. Mater..

[cit98] Guo Z., Gao L., Xu Z., Teo S., Zhang C., Kamata Y., Hayase S., Ma T. (2018). Small.

[cit99] Ronchi R. M., Arantes J. T., Santos S. F. (2019). Ceram. Int..

[cit100] Chakraborty P., Das T., Saha-Dasgupta T. (2019). Compr. Nanosci. Nanotechnol..

[cit101] Manikandan A., Chen Y. Z., Shen C. C., Sher C. W., Kuo H. C., Chueh Y. L. (2019). Prog. Quantum Electron..

[cit102] Liu X. X., Zhang Z., Jiang J., Tian C., Wang X., Wang L., Zhang Z., Wu X., Zheng Y., Liang J., Chen C. C. (2022). Chem. Eng. J..

[cit103] Zhao X., Radovic M., Green M. J. (2020). Chem.

[cit104] Chen X., Li J., Pan G., Xu W., Zhu J., Zhou D., Li D., Chen C., Lu G., Song H. (2019). Sens. Actuators, B.

[cit105] Al-Duais M. A., Mohammedsaleh Z. M., Al-Shehri H. S., Al-Awthan Y. S., Bani-Atta S. A., Keshk A. A., Mustafa S. K., Althaqafy A. D., Al-Tweher J. N., Al-Aoh H. A., Panneerselvam C. (2022). Luminescence.

[cit106] Zhang T., Deng F., Shi P. (2023). IEEE Trans. Autom. Control.

[cit107] Dai C., Lin H., Xu G., Liu Z., Wu R., Chen Y. (2017). Chem. Mater..

[cit108] Cai Y., Shen J., Ge G., Zhang Y., Jin W., Huang W., Dong X. (2018). ACS Nano.

[cit109] Han R., Ma X., Xie Y., Teng D., Zhang S. (2017). RSC Adv..

[cit110] Boota M., Pasini M., Galeotti F., Porzio W., Zhao M., Halim J., Gogotsi Y. (2017). Chem. Mater..

[cit111] Khosla A., Awan H., Singh K., Walvekar R., Chaudhary V. (2022). Adv. Sci..

[cit112] El-Denglawey A., Angadi V., Manjunatha K., Chethan B., Somvanshi S. (2021). J. Mater. Sci.: Mater. Electron..

[cit113] Drozd D. D., Strokin P. D., Kornilov D. A., Drozd A. V., Burov A. M., Ushakov A. V., Goryacheva O. A., Goryacheva I. Yu. (2024). Chem. Eng. J..

[cit114] Tiwari P., Sahu M., Kumar G., Ashourian M. (2021). Comput. Intell. Neurosci..

[cit115] Zhu Y., Feng L., Zhao R., Liu B., Yang P. (2024). ACS Appl. Nano Mater..

[cit116] Vénosová B., Karlický F. (2023). Nanoscale Adv..

[cit117] Nguyen T. N. A., Doong R. A., Liu K. K. (2025). Microchem. J..

[cit118] Yu L., Feng L., Xiong L., Li S., Wang S., Wei Z., Xiao Y. (2022). J. Hazard. Mater..

[cit119] Zhang P. P., Ni A. Y., Zhang J. J., Zhang B. L., Zhou H. A., Zhao H., Liu S., Ni J., Duan C. (2023). Sens. Actuators, B.

[cit120] Han L., Dong X. Z., Liu S. G., Wang X. H., Ling Y., Li N. B., Luo H. Q. (2023). Environ. Sci.: Nano.

[cit121] Niu X., Wang M., Zhang M., Cao R., Liu Z., Hao F., Sheng L., Xu H. (2022). Inorg. Chem. Front..

[cit122] Lin C., Qiu C., Wang Y., Liu Y., Rong M., Niu L. (2024). Sens. Diagn..

[cit123] Wang Q., Wen X., Kong J. (2020). Crit. Rev. Anal. Chem..

[cit124] Maiuolo J., Oppedisano F., Gratteri S., Muscoli C., Mollace V. (2016). Int. J. Cardiol..

[cit125] Liu M., Wang Y., Tang S., Wang W., Liang A., Luo A. (2024). Bioelectrochemistry.

[cit126] Duan F., Guo C., Hu M., Song Y., Wang M., He L., Zhang Z., Pettinari R., Zhou L. (2020). Sens. Actuators, B.

[cit127] Naik K., Chaudhary S., Ye L., Parmar A. S. (2022). Front. Bioeng. Biotechnol..

[cit128] Sato K., Suematsu A., Okamoto K., Yamaguchi A., Morishita Y., Kadono Y., Tanaka S., Kodama T., Akira S., Iwakura Y., Cua D. J. (2006). J. Exp. Med..

[cit129] Rafieerad A., Yan W., Sequiera G. L., Sareen N., Abu-El-Rub E., Moudgil M., Dhingra S. (2019). Adv. Healthcare Mater..

[cit130] Liu G., Zou J., Tang Q., Yang X., Zhang Y., Zhang Q., Huang W., Chen P., Shao J., Dong X. (2017). ACS Appl. Mater. Interfaces.

[cit131] Yu Z., Chan W. K., Zhang Y., Tan T. T. Y. (2021). Biomaterials.

[cit132] Li L., Xing Z., Liao T., Wang J., Xu Z., Kuang Y., Li C. (2024). Mater. Today Chem..

[cit133] Won S. Y., Singhmar R., Sahoo S., Kim H., Kim C. M., Choi S. M., Sood A., Han S. S. (2025). Colloids Surf., B.

[cit134] Hu B., Chen J., Gao Z., Chen L., Cao T., Li H., Yu Q., Wang C., Gan Z. (2024). ACS Appl. Bio Mater..

[cit135] Nie Y., Liang Z., Wang P., Ma Q., Su X. (2021). Anal. Chem..

[cit136] Li X., Liu F., Huang D., Xue N., Dang Y., Zhang M., Zhang L., Li B., Liu D., Wang L., Liu H. (2020). Adv. Funct. Mater..

[cit137] Bitounis D., Ali-Boucetta H., Hong B., Min D., Kostarelos K. (2013). Adv. Mater..

[cit138] Won S., Singhmar R., Sahoo S., Kim H., Kim C., Choi S., Han S. (2025). Colloids Surf., B.

[cit139] Kulkarni S., Soman S., Navti P., Roy A., Nikam A., Vineeth P., Mutalik S. (2024). Materials.

[cit140] Ruzycka-Ayoush M., Kowalik P., Kowalczyk A., Bujak P., Nowicka A. M., Wojewodzka M., Kruszewski M., Grudzinski I. P. (2021). Cancer Nanotechnol..

[cit141] Johari-Ahar M., Barar J., Alizadeh A. M., Davaran S., Omidi Y., Rashidi M. R. (2016). J. Drug Targeting.

[cit142] Pandey S., Thakur M., Mewada A., Anjarlekar D., Mishra N., Sharon M. (2013). J. Mater. Chem. B.

[cit143] Ghafary S. M., Rahimjazi E., Hamzehil H., Mousavi S. M. M., Nikkhah M., Hosseinkhani S. (2022). Nanomed. Nanotechnol. Biol. Med..

[cit144] Cao Y., Wu T., Zhang K., Meng X., Dai W., Wang D., Dong H., Zhang X. (2019). ACS Nano.

[cit145] Du L., Zhang H., Zhang F., Xia J., Meng Q., Huang H., Wang Z. (2024). Talanta.

[cit146] Bai Y., He Y., Wang Y., Song G. (2021). Microchim. Acta.

[cit147] Chen F., Gerion D. (2004). Nano Lett..

[cit148] Bagyalakshmi S., Thilakavathi G., Sivakami A., Sukumar T., Gowri S. (2026). Mater. Sci. Semicond. Process..

[cit149] Abu N., Chinnathambi S., Kumar M., Etezadi F., Bakhori N., Zubir Z., Pandian G. (2023). RSC Adv..

[cit150] Yi C., Yu Z., Ren Q., Liu X., Wang Y., Sun X., Huang X. (2020). Photodiagn. Photodyn. Ther..

[cit151] Barkan N. P. , Turan S. K., Yildizhan H., Demiralp F. D. Ö., Uslu B. and Ozkan S. A., in Design of Nanostructures for Theranostics Applications, William Andrew Publishing, 2018, pp. 493–528

[cit152] Merum S., Veluru J., Seeram R. (2017). Mater. Sci. Eng., B.

[cit153] Wang X., Barmin R., Pallares R. M., Rix A., Kiessling F. (2026). Acta Pharm. Sin. B.

[cit154] Beringhs A. O. R. , Sadabad R. K. and Lu X., in Biomaterials for Cancer Therapeutics, Woodhead Publishing, 2020, pp. 291–329

[cit155] Farani M., Mirzaee D., Hatami A., Kumar K., Ghoreishian S., Huh Y. (2026). Bioact. Mater..

[cit156] Rafieerad A., Yan W., Amiri A., Dhingra S. (2020). Mater. Des..

[cit157] Wei H., Dong J., Fang X., Zheng W., Sun Y., Qian Y., Huang Y. (2019). Compos. Sci. Technol..

[cit158] Xiao P., Gu J., Chen J., Zhang J., Xing R., Han Y., Chen T. (2014). Chem. Commun..

[cit159] Xiao Z. , Ruan S., Kong L. B., Que W., Zhou K., Liu Y. and Zhang T., MXenes and MXene-Based Composites, Springer International Publishing, Cham, 2020

[cit160] You Q., Wang P., Zhu T., Jia Z., Chang Z., Li L., Dong W. F. (2025). Mater. Today Bio.

[cit161] Zhou L., Wu F., Yu J., Deng Q., Zhang F., Wang G. (2017). Carbon.

[cit162] Luo W., Liu H., Liu X., Liu L., Zhao W. (2021). Colloids Surf., B.

[cit163] Yan X., Ma J., Yu K., Li J., Yang L., Liu J., Wang J., Cai L. (2020). Chin. Chem. Lett..

[cit164] Zhao L., Wang L., Zheng Y., Zhao S., Wei W., Zhang D., Fu X., Jiang K., Shen G., Han W. (2021). Nano Energy.

[cit165] Amani A. M., Tayebi L., Abbasi M., Vaez A., Kamyab H., Chelliapan S., Vafa E. (2023). ACS Omega.

[cit166] Chandran M., Chellasamy G., Veerapandian M., Dhanasekaran B., Govindaraju S., Yun K. (2025). J. Mater. Chem. B.

[cit167] Zheng Y., Wang Y., Li Z., Yuan Z., Guo S., Lou Z., Han W., Shen G., Wang L. (2023). Matter.

[cit168] Li M., Li Z., Wang P., Ma Q. (2023). Biosens. Bioelectron..

[cit169] Shenoy N., Creagan E., Witzig T., Levine M. (2018). Cancer Cell.

[cit170] Cheng H., Li L., Zhang M., Jiang Y., Yu P., Ma F., Mao L. (2018). TrAC, Trends Anal. Chem..

[cit171] Sampaio I., Quatroni F. D., Zucolotto V. (2020). Alzheimer's Dementia.

[cit172] Hu Y., He Y., Peng Z., Li Y. (2020). Biosens. Bioelectron..

[cit173] Han Y., Quan K., Chen J., Qiu H. (2020). Biosens. Bioelectron..

[cit174] Hu Y., Wang Q., Yu J., Zhou Q., Deng Y., Liu J., Zhang L., Xu Y., Xiong W., Wang Y. (2022). Nat. Commun..

[cit175] Kong H. Y., Byun J. (2013). Biomol. Ther..

[cit176] Pfeiffer M., Crean R. M., Moreira C., Parracino A., Oberdorfer G., Brecker L., Hammerschmidt F., Kamerlin S. C. L., Nidetzky B. (2022). ACS Catal..

[cit177] Li L., Ge J., Wu H., Xu Q. H., Yao S. Q. (2012). J. Am. Chem. Soc..

[cit178] Ren D., Cheng X., Chen Q., Xu G., Wei F., Yang J., Xu J., Wang L., Hu Q., Cen Y. (2023). Microchem. J..

[cit179] Rafieerad A., Yan W., Alagarsamy K. N., Srivastava A., Sareen N., Arora R. C., Dhingra S. (2021). Adv. Funct. Mater..

[cit180] Liu Y., Lv Y., Chen W., Yang X., Cheng X., Rong Z., Wang S. (2023). ACS Appl. Mater. Interfaces.

[cit181] Paul J., Kim J. (2024). ACS Appl. Nano Mater..

[cit182] Zhang F., Sun G., Zhao R., Yang F., Jiang X., Song S., Zhang J., Shen H., Shen J. (2024). Langmuir.

[cit183] Fu C., Ai F., Huang J., Shi Z., Yan X., Zheng X. (2022). Spectrochim. Acta, Part A.

[cit184] Ahmed S., Ning J., Peng D., Chen T., Ahmad I., Ali A., Lei Z., Shabbir M., Cheng G., Yuan Z. (2020). Food Agric. Immunol..

[cit185] Mahe R., Ye Z., Qi M., Cai W., Wen Y., Xiao J., Zhao J. (2024). Rev. Fish. Sci. Aquac..

[cit186] Yao H., Pugliese D., Lancry M., Dai Y. (2024). Laser Photonics Rev..

[cit187] Zhang H., Wang P., Liu Y., Dai S., Zhu Y., Li B., Dong G. (2024). ACS Appl. Mater. Interfaces.

[cit188] Zhou Y., Zhang Y., Ruan K., Guo H., He M., Qiu H., Gu J. (2024). Sci. Bull..

[cit189] Awasthi G., Maharjan B., Shrestha S., Bhattarai D., Yoon D., Park C., Kim C. (2020). Colloids Surf., A.

[cit190] Karami M., Moini Jazani O. (2026). Discover Nano.

[cit191] Xu S., Du C., Zhang M., Wang R., Feng W., Wang C., Zhao W. (2023). Nano Res..

[cit192] Demir H., Balay H., Kirnap M. (2004). J. Rehabil. Res. Dev..

[cit193] Zanganeh M., Moghim I. (2023). Adv. Appl. NanoBio-Technol..

